# Supplementary material for: A Sequel to Sanger: amplicon sequencing that scales
Source: BMC Genomics. 2018 Mar 27;19:219. doi: 10.1186/s12864-018-4611-3 (PMC5870082; doi:10.1186/s12864-018-4611-3)
Supplement: Supplementary file 1 — Figure S1. Sliding window (5 bp) analysis showing the distribution of homopolymer regions in the COI gene for the 95 taxa in library #1, the GC composition of the homopolymer regions, the incidence and position of indels in and substitutions in COI sequences for each window in the 99.9% SMRT partition. The incidence of substitutions and indels are shown per base pair. Figure S2. Bivariate plot showing the mean number of indels per CCS for each taxon in the four libraries versus the GC content of a COI amplicon. The number of indels was determined through comparison with the corresponding Sanger sequence for each taxon. Figure S3. Bivariate plot showing the mean number of indels in SMRT sequences for the 95 specimens shared by Libraries #1 and #2. Figure S4. NJ tree of COI sequences for the 945 species in Library #2 showing the correspondence between Sanger and SMRT sequences. Figure S5. Distribution of similarity values between Sanger and SMRT sequences for the 9120 specimens in Library #3 and the 9830 specimens in Library #4 for the three SMRT partitions. Figure S6. Relationship between the number of circular consensus sequences for a COI amplicon and its GC content. The 95 and 950 amplicon datasets (Libraries #1/2) were generated via the PCR1 protocol while the 9120 and 9830 amplicon datasets (Libraries #3/4) were generated via the PCR2 protocol. Figure S7. Incidence of low quality Sanger reads in cases of target and non-target wells for Libraries #3/4. (PDF 4664 kb) [file 12864_2018_4611_MOESM1_ESM.pdf]

Additional File 1

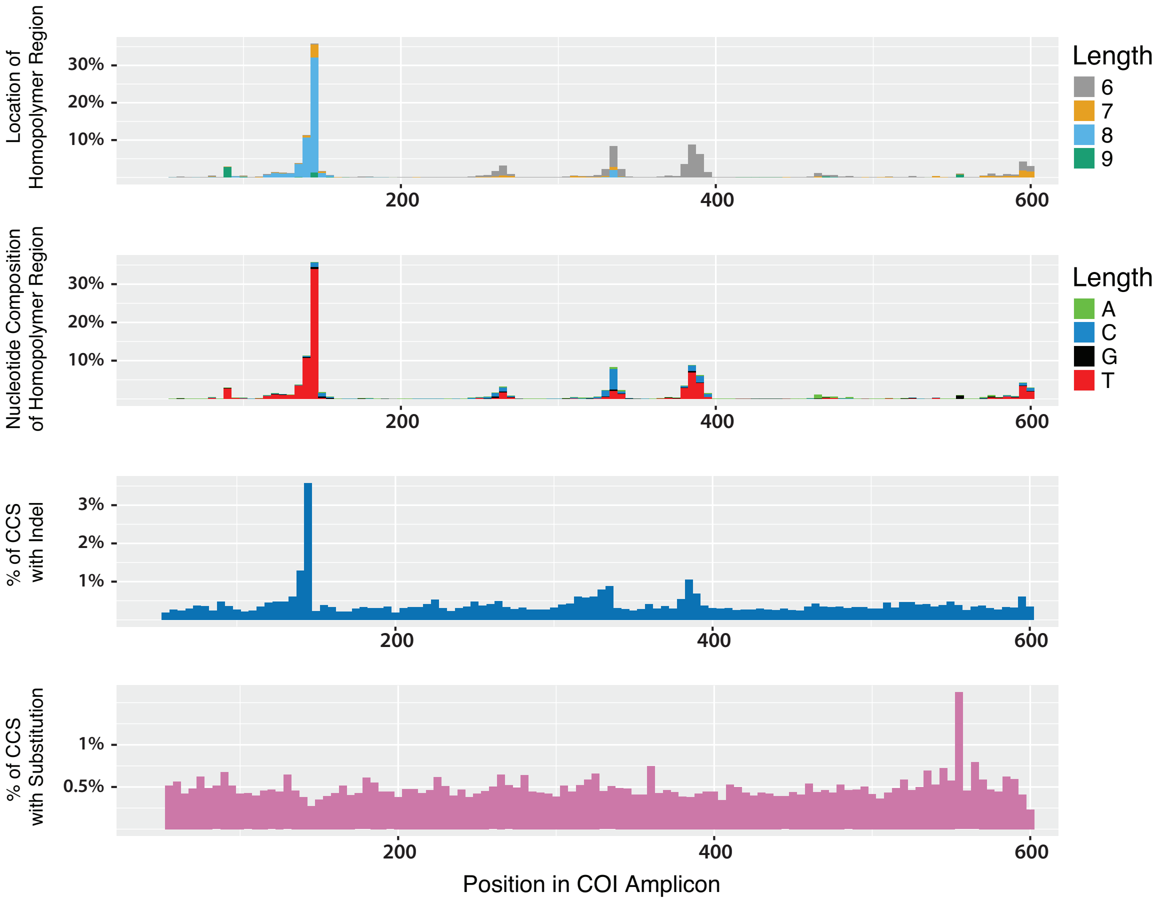

Figure S1. Sliding window (5 bp) analysis showing the distribution of homopolymer regions in the COI gene for the 95 taxa in library #1, the GC composition of the homopolymer regions, the incidence and position of indels in and substitutions in COI sequences for each window in the 99.9% SMRT partition. The incidence of substitutions and indels are shown per base pair.

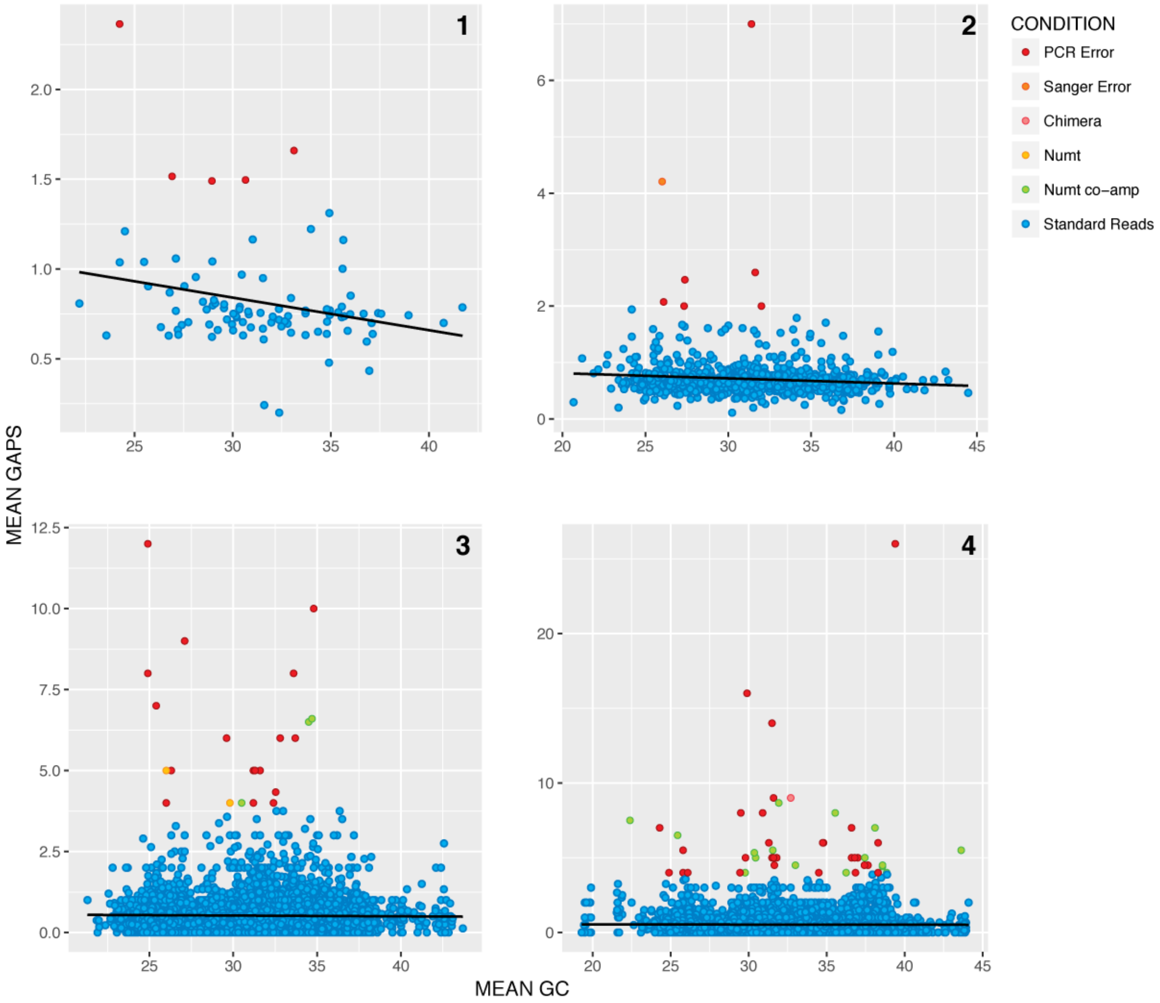

Figure S2. Bivariate plot showing the mean number of indels per CCS for each taxon in the four libraries versus the GC content of its COI amplicon. The number of indels is determined through comparison with the corresponding Sanger sequence for each taxon.

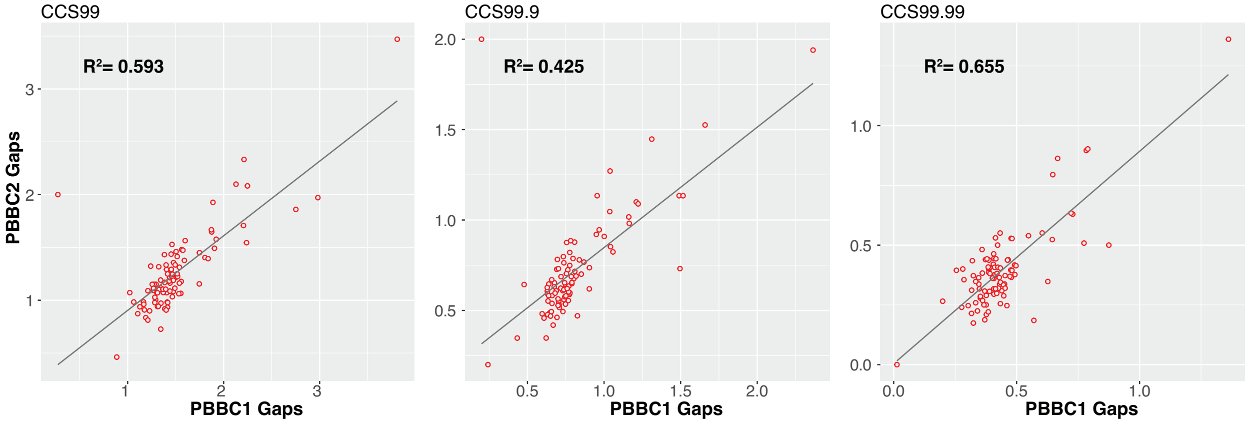

Figure S3. Bivariate plot showing the mean number of indels in SMRT sequences for the 95 specimens that were shared by Libraries #1 and #2.

Figure S4. NJ tree of COI sequences for the 945 species in Library #2 showing the correspondence between Sanger and SMRT sequences. (19pp.)

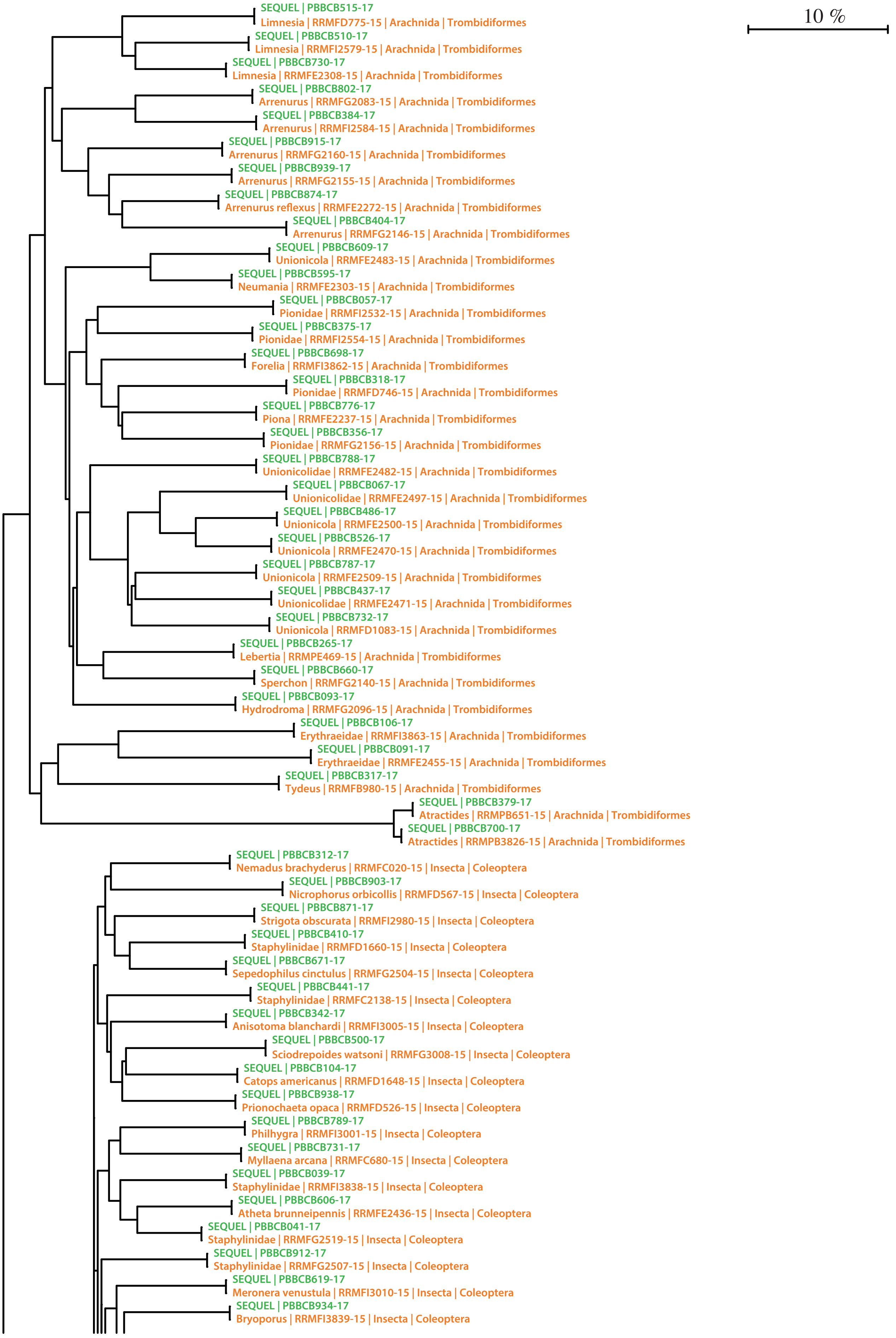

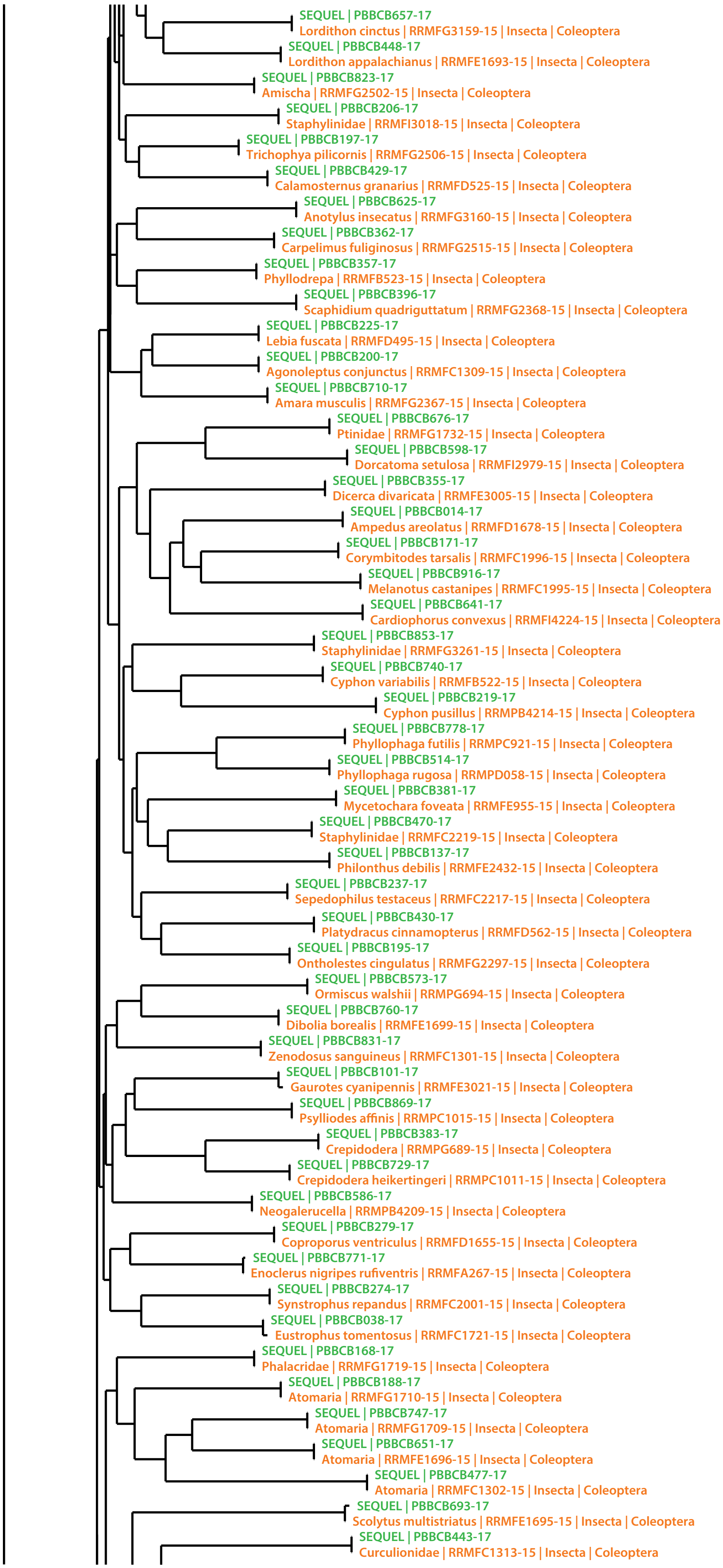
SEQUEL | PBBCB657-17
Lordithon cinctus | RRMFG3159-15 | Insecta | Coleoptera
SEQUEL | PBBCB448-17
Lordithon appalachianus | RRMFE1693-15 | Insecta | Coleoptera
SEQUEL | PBBCB823-17
Amischa | RRMFG2502-15 | Insecta | Coleoptera
SEQUEL | PBBCB206-17
Staphylinidae | RRMFI3018-15 | Insecta | Coleoptera
SEQUEL | PBBCB197-17
Trichophya pilicornis | RRMFG2506-15 | Insecta | Coleoptera
SEQUEL | PBBCB429-17
Calamosternus granarius | RRMFD525-15 | Insecta | Coleoptera
SEQUEL | PBBCB625-17
Anotylus insecatus | RRMFG3160-15 | Insecta | Coleoptera
SEQUEL | PBBCB362-17
Carpelimus fuliginosus | RRMFG2515-15 | Insecta | Coleoptera
SEQUEL | PBBCB357-17
Phyllodrepa | RRMFB523-15 | Insecta | Coleoptera
SEQUEL | PBBCB396-17
Scaphidium quadriguttatum | RRMFG2368-15 | Insecta | Coleoptera
SEQUEL | PBBCB225-17
Lebia fuscata | RRMFD495-15 | Insecta | Coleoptera
SEQUEL | PBBCB200-17
Agonoleptus conjunctus | RRMFC1309-15 | Insecta | Coleoptera
SEQUEL | PBBCB710-17
Amara musculis | RRMFG2367-15 | Insecta | Coleoptera
SEQUEL | PBBCB676-17
Ptinidae | RRMFG1732-15 | Insecta | Coleoptera
SEQUEL | PBBCB598-17
Dorcatoma setulosa | RRMFI2979-15 | Insecta | Coleoptera
SEQUEL | PBBCB355-17
Dicerca divaricata | RRMFE3005-15 | Insecta | Coleoptera
SEQUEL | PBBCB014-17
Ampedus areolatus | RRMFD1678-15 | Insecta | Coleoptera
SEQUEL | PBBCB171-17
Corymbitodes tarsalis | RRMFC1996-15 | Insecta | Coleoptera
SEQUEL | PBBCB916-17
Melanotus castanipes | RRMFC1995-15 | Insecta | Coleoptera
SEQUEL | PBBCB641-17
Cardiophorus convexus | RRMFI4224-15 | Insecta | Coleoptera
SEQUEL | PBBCB853-17
Staphylinidae | RRMFG3261-15 | Insecta | Coleoptera
SEQUEL | PBBCB740-17
Cyphon variabilis | RRMFB522-15 | Insecta | Coleoptera
SEQUEL | PBBCB219-17
Cyphon pusillus | RRMPB4214-15 | Insecta | Coleoptera
SEQUEL | PBBCB778-17
Phyllophaga futilis | RRMPC921-15 | Insecta | Coleoptera
SEQUEL | PBBCB514-17
Phyllophaga rugosa | RRMPD058-15 | Insecta | Coleoptera
SEQUEL | PBBCB381-17
Mycetochara foveata | RRMFE955-15 | Insecta | Coleoptera
SEQUEL | PBBCB470-17
Staphylinidae | RRMFC2219-15 | Insecta | Coleoptera
SEQUEL | PBBCB137-17
Philonthus debilis | RRMFE2432-15 | Insecta | Coleoptera
SEQUEL | PBBCB237-17
Sepedophilus testaceus | RRMFC2217-15 | Insecta | Coleoptera
SEQUEL | PBBCB430-17
Platydracus cinnamopterus | RRMFD562-15 | Insecta | Coleoptera
SEQUEL | PBBCB195-17
Ontholestes cingulatus | RRMFG2297-15 | Insecta | Coleoptera
SEQUEL | PBBCB573-17
Ormiscus walshii | RRMPG694-15 | Insecta | Coleoptera
SEQUEL | PBBCB760-17
Dibolia borealis | RRMFE1699-15 | Insecta | Coleoptera
SEQUEL | PBBCB831-17
Zenodosus sanguineus | RRMFC1301-15 | Insecta | Coleoptera
SEQUEL | PBBCB101-17
Gaurotes cyanipennis | RRMFE3021-15 | Insecta | Coleoptera
SEQUEL | PBBCB869-17
Psylliodes affinis | RRMPC1015-15 | Insecta | Coleoptera
SEQUEL | PBBCB383-17
Crepidodera | RRMPG689-15 | Insecta | Coleoptera
SEQUEL | PBBCB729-17
Crepidodera heikertingeri | RRMPC1011-15 | Insecta | Coleoptera
SEQUEL | PBBCB586-17
Neogalerucella | RRMPB4209-15 | Insecta | Coleoptera
SEQUEL | PBBCB279-17
Coproporus ventriculus | RRMFD1655-15 | Insecta | Coleoptera
SEQUEL | PBBCB771-17
Enoclerus nigripes rufiventris | RRMFA267-15 | Insecta | Coleoptera
SEQUEL | PBBCB274-17
Synstrophus repandus | RRMFC2001-15 | Insecta | Coleoptera
SEQUEL | PBBCB038-17
Eustrophus tomentosus | RRMFC1721-15 | Insecta | Coleoptera
SEQUEL | PBBCB168-17
Phalacridae | RRMFG1719-15 | Insecta | Coleoptera
SEQUEL | PBBCB188-17
Atomaria | RRMFG1710-15 | Insecta | Coleoptera
SEQUEL | PBBCB747-17
Atomaria | RRMFG1709-15 | Insecta | Coleoptera
SEQUEL | PBBCB651-17
Atomaria | RRMFE1696-15 | Insecta | Coleoptera
SEQUEL | PBBCB477-17
Atomaria | RRMFC1302-15 | Insecta | Coleoptera
SEQUEL | PBBCB693-17
Scolytus multistriatus | RRMFE1695-15 | Insecta | Coleoptera
SEQUEL | PBBCB443-17
Curculionidae | RRMFC1313-15 | Insecta | Coleoptera

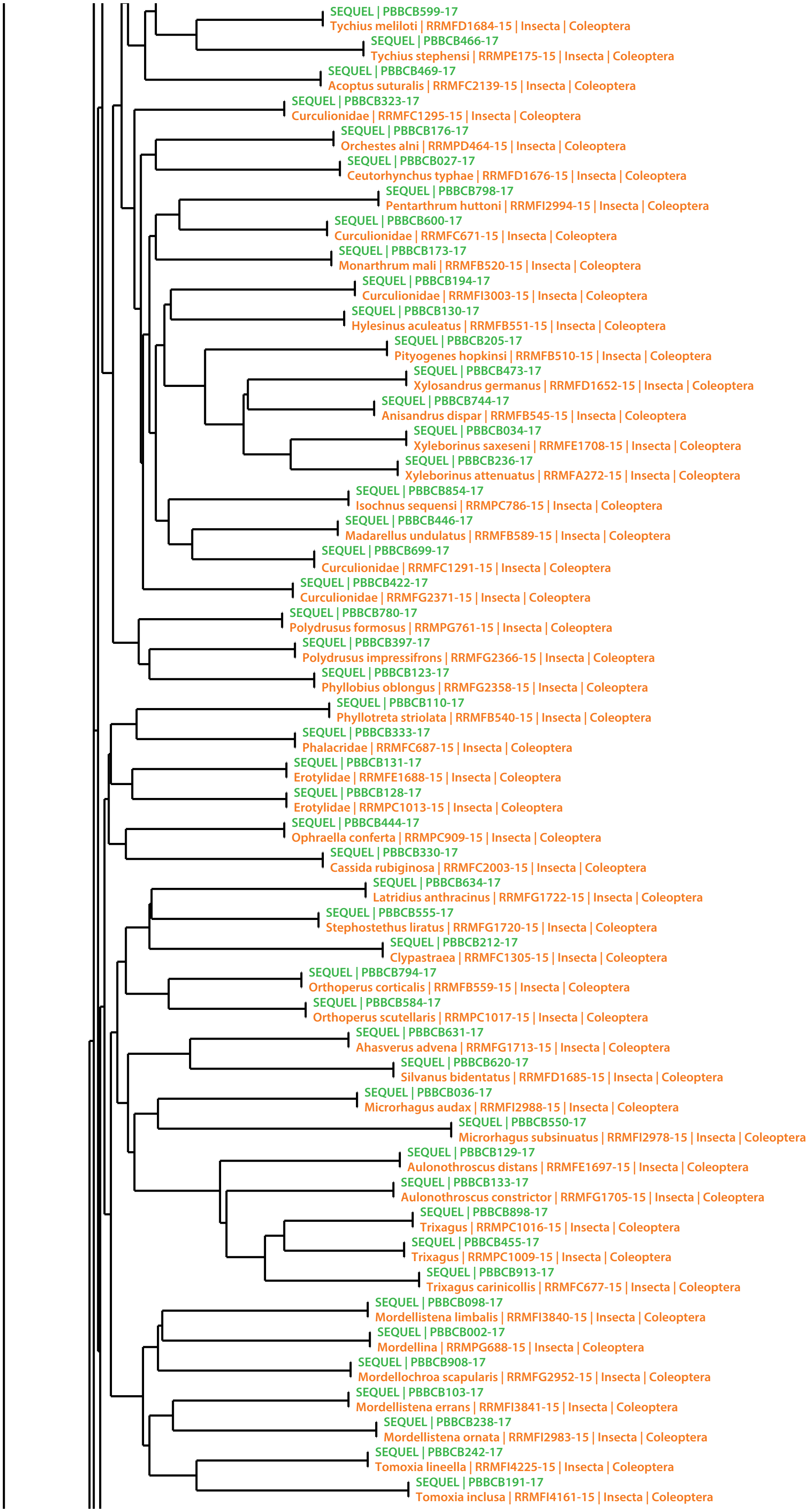
SEQUEL | PBBCB599-17
Tychius meliloti | RRMFD1684-15 | Insecta | Coleoptera
SEQUEL | PBBCB466-17
Tychius stephensi | RRMPE175-15 | Insecta | Coleoptera
SEQUEL | PBBCB469-17
Acoptus suturalis | RRMFC2139-15 | Insecta | Coleoptera
SEQUEL | PBBCB323-17
Curculionidae | RRMFC1295-15 | Insecta | Coleoptera
SEQUEL | PBBCB176-17
Orchestes alni | RRMPD464-15 | Insecta | Coleoptera
SEQUEL | PBBCB027-17
Ceutorhynchus typhae | RRMFD1676-15 | Insecta | Coleoptera
SEQUEL | PBBCB798-17
Pentarthrum huttoni | RRMFI2994-15 | Insecta | Coleoptera
SEQUEL | PBBCB600-17
Curculionidae | RRMFC671-15 | Insecta | Coleoptera
SEQUEL | PBBCB173-17
Monarthrum mali | RRMFB520-15 | Insecta | Coleoptera
SEQUEL | PBBCB194-17
Curculionidae | RRMFI3003-15 | Insecta | Coleoptera
SEQUEL | PBBCB130-17
Hylesinus aculeatus | RRMFB551-15 | Insecta | Coleoptera
SEQUEL | PBBCB205-17
Pityogenes hopkinsi | RRMFB510-15 | Insecta | Coleoptera
SEQUEL | PBBCB473-17
Xylosandrus germanus | RRMFD1652-15 | Insecta | Coleoptera
SEQUEL | PBBCB744-17
Anisandrus dispar | RRMFB545-15 | Insecta | Coleoptera
SEQUEL | PBBCB034-17
Xyleborinus saxeseni | RRMFE1708-15 | Insecta | Coleoptera
SEQUEL | PBBCB236-17
Xyleborinus attenuatus | RRMFA272-15 | Insecta | Coleoptera
SEQUEL | PBBCB854-17
Isochnus sequensi | RRMPC786-15 | Insecta | Coleoptera
SEQUEL | PBBCB446-17
Madarellus undulatus | RRMFB589-15 | Insecta | Coleoptera
SEQUEL | PBBCB699-17
Curculionidae | RRMFC1291-15 | Insecta | Coleoptera
SEQUEL | PBBCB422-17
Curculionidae | RRMFG2371-15 | Insecta | Coleoptera
SEQUEL | PBBCB780-17
Polydrusus formosus | RRMPG761-15 | Insecta | Coleoptera
SEQUEL | PBBCB397-17
Polydrusus impressifrons | RRMFG2366-15 | Insecta | Coleoptera
SEQUEL | PBBCB123-17
Phyllobius oblongus | RRMFG2358-15 | Insecta | Coleoptera
SEQUEL | PBBCB110-17
Phyllotreta striolata | RRMFB540-15 | Insecta | Coleoptera
SEQUEL | PBBCB333-17
Phalacridae | RRMFC687-15 | Insecta | Coleoptera
SEQUEL | PBBCB131-17
Erotylidae | RRMFE1688-15 | Insecta | Coleoptera
SEQUEL | PBBCB128-17
Erotylidae | RRMPC1013-15 | Insecta | Coleoptera
SEQUEL | PBBCB444-17
Ophraella conferta | RRMPC909-15 | Insecta | Coleoptera
SEQUEL | PBBCB330-17
Cassida rubiginosa | RRMFC2003-15 | Insecta | Coleoptera
SEQUEL | PBBCB634-17
Latridius anthracinus | RRMFG1722-15 | Insecta | Coleoptera
SEQUEL | PBBCB555-17
Stephostethus liratus | RRMFG1720-15 | Insecta | Coleoptera
SEQUEL | PBBCB212-17
Clypastraea | RRMFC1305-15 | Insecta | Coleoptera
SEQUEL | PBBCB794-17
Orthoperus corticalis | RRMFB559-15 | Insecta | Coleoptera
SEQUEL | PBBCB584-17
Orthoperus scutellaris | RRMPC1017-15 | Insecta | Coleoptera
SEQUEL | PBBCB631-17
Ahasverus advena | RRMFG1713-15 | Insecta | Coleoptera
SEQUEL | PBBCB620-17
Silvanus bidentatus | RRMFD1685-15 | Insecta | Coleoptera
SEQUEL | PBBCB036-17
Microrhagus audax | RRMFI2988-15 | Insecta | Coleoptera
SEQUEL | PBBCB550-17
Microrhagus subsinuatus | RRMFI2978-15 | Insecta | Coleoptera
SEQUEL | PBBCB129-17
Aulonothroscus distans | RRMFE1697-15 | Insecta | Coleoptera
SEQUEL | PBBCB133-17
Aulonothroscus constrictor | RRMFG1705-15 | Insecta | Coleoptera
SEQUEL | PBBCB898-17
Trixagus | RRMPC1016-15 | Insecta | Coleoptera
SEQUEL | PBBCB455-17
Trixagus | RRMPC1009-15 | Insecta | Coleoptera
SEQUEL | PBBCB913-17
Trixagus carinicollis | RRMFC677-15 | Insecta | Coleoptera
SEQUEL | PBBCB098-17
Mordellistena limbalis | RRMFI3840-15 | Insecta | Coleoptera
SEQUEL | PBBCB002-17
Mordellina | RRMPG688-15 | Insecta | Coleoptera
SEQUEL | PBBCB908-17
Mordellochroa scapularis | RRMFG2952-15 | Insecta | Coleoptera
SEQUEL | PBBCB103-17
Mordellistena errans | RRMFI3841-15 | Insecta | Coleoptera
SEQUEL | PBBCB238-17
Mordellistena ornata | RRMFI2983-15 | Insecta | Coleoptera
SEQUEL | PBBCB242-17
Tomoxia lineella | RRMFI4225-15 | Insecta | Coleoptera
SEQUEL | PBBCB191-17
Tomoxia inclusa | RRMFI4161-15 | Insecta | Coleoptera

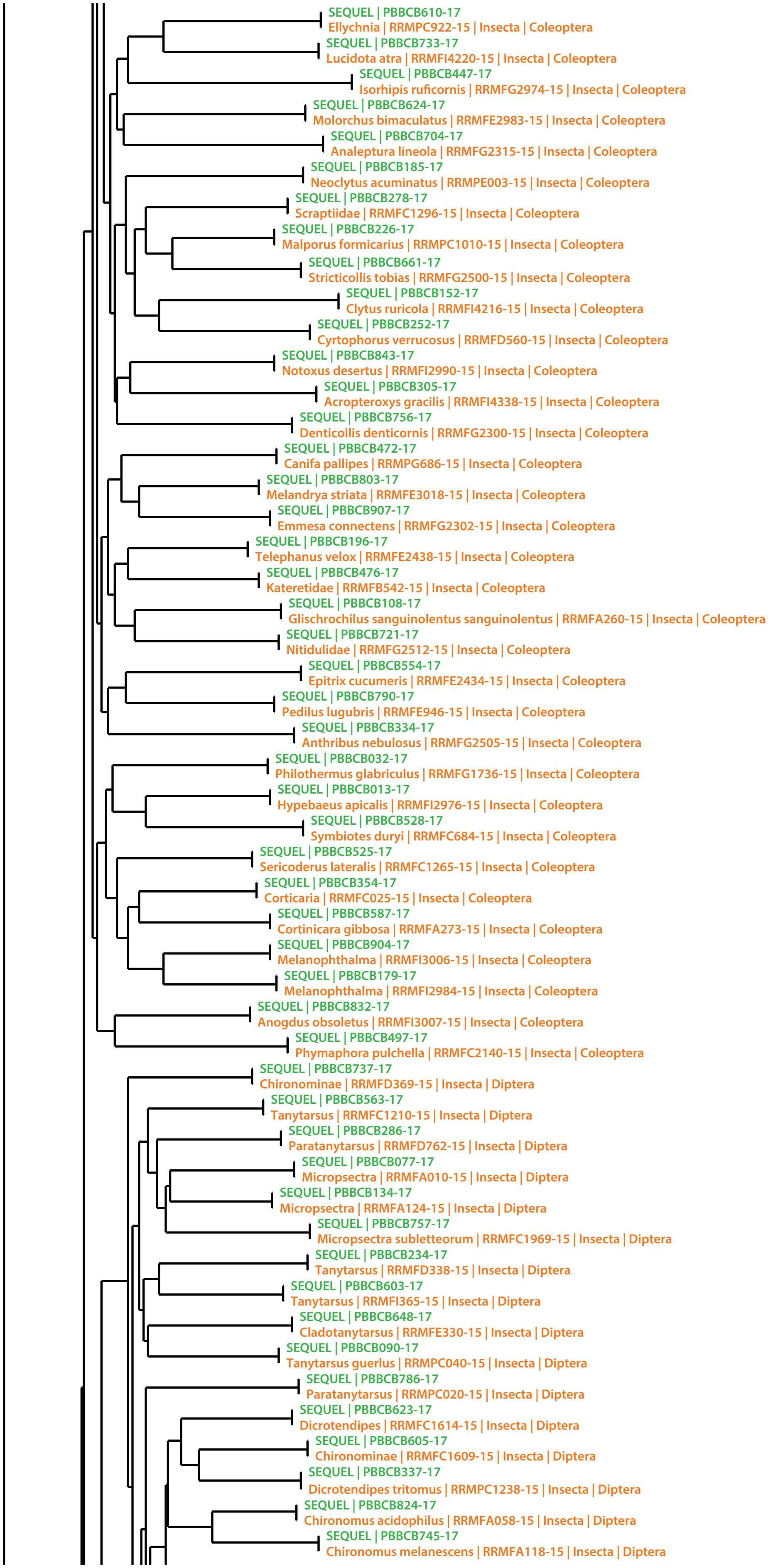
SEQUEL | PBBCB610-17
Ellychnia | RRMPC922-15 | Insecta | Coleoptera
SEQUEL | PBBCB733-17
Lucidota atra | RRMFI4220-15 | Insecta | Coleoptera
SEQUEL | PBBCB447-17
Isorhipis ruficornis | RRMFG2974-15 | Insecta | Coleoptera
SEQUEL | PBBCB624-17
Molorchus bimaculatus | RRMFE2983-15 | Insecta | Coleoptera
SEQUEL | PBBCB704-17
Analeptura lineola | RRMFG2315-15 | Insecta | Coleoptera
SEQUEL | PBBCB185-17
Neoclytus acuminatus | RRMPE003-15 | Insecta | Coleoptera
SEQUEL | PBBCB278-17
Scraptiidae | RRMFC1296-15 | Insecta | Coleoptera
SEQUEL | PBBCB226-17
Malporus formicarius | RRMPC1010-15 | Insecta | Coleoptera
SEQUEL | PBBCB661-17
Stricticollis tobias | RRMFG2500-15 | Insecta | Coleoptera
SEQUEL | PBBCB152-17
Clytus ruricola | RRMFI4216-15 | Insecta | Coleoptera
SEQUEL | PBBCB252-17
Cyrtophorus verrucosus | RRMFD560-15 | Insecta | Coleoptera
SEQUEL | PBBCB843-17
Notoxus desertus | RRMFI2990-15 | Insecta | Coleoptera
SEQUEL | PBBCB305-17
Acropteroxys gracilis | RRMFI4338-15 | Insecta | Coleoptera
SEQUEL | PBBCB756-17
Denticollis denticornis | RRMFG2300-15 | Insecta | Coleoptera
SEQUEL | PBBCB472-17
Canifa pallipes | RRMPG686-15 | Insecta | Coleoptera
SEQUEL | PBBCB803-17
Melandrya striata | RRMFE3018-15 | Insecta | Coleoptera
SEQUEL | PBBCB907-17
Emmesa connectens | RRMFG2302-15 | Insecta | Coleoptera
SEQUEL | PBBCB196-17
Telephanus velox | RRMFE2438-15 | Insecta | Coleoptera
SEQUEL | PBBCB476-17
Kateretidae | RRMFB542-15 | Insecta | Coleoptera
SEQUEL | PBBCB108-17
Glischrochilus sanguinolentus sanguinolentus | RRMFA260-15 | Insecta | Coleoptera
SEQUEL | PBBCB721-17
Nitidulidae | RRMFG2512-15 | Insecta | Coleoptera
SEQUEL | PBBCB554-17
Epitrix cucumeris | RRMFE2434-15 | Insecta | Coleoptera
SEQUEL | PBBCB790-17
Pedilus lugubris | RRMFE946-15 | Insecta | Coleoptera
SEQUEL | PBBCB334-17
Anthribus nebulosus | RRMFG2505-15 | Insecta | Coleoptera
SEQUEL | PBBCB032-17
Philothermus glabriculus | RRMFG1736-15 | Insecta | Coleoptera
SEQUEL | PBBCB013-17
Hypebaeus apicalis | RRMFI2976-15 | Insecta | Coleoptera
SEQUEL | PBBCB528-17
Symbiotes duryi | RRMFC684-15 | Insecta | Coleoptera
SEQUEL | PBBCB525-17
Sericoderus lateralis | RRMFC1265-15 | Insecta | Coleoptera
SEQUEL | PBBCB354-17
Corticaria | RRMFC025-15 | Insecta | Coleoptera
SEQUEL | PBBCB587-17
Cortinicara gibbosa | RRMFA273-15 | Insecta | Coleoptera
SEQUEL | PBBCB904-17
Melanophthalma | RRMFI3006-15 | Insecta | Coleoptera
SEQUEL | PBBCB179-17
Melanophthalma | RRMFI2984-15 | Insecta | Coleoptera
SEQUEL | PBBCB832-17
Anogdus obsoletus | RRMFI3007-15 | Insecta | Coleoptera
SEQUEL | PBBCB497-17
Phymaphora pulchella | RRMFC2140-15 | Insecta | Coleoptera
SEQUEL | PBBCB737-17
Chironominae | RRMFD369-15 | Insecta | Diptera
SEQUEL | PBBCB563-17
Tanytarsus | RRMFC1210-15 | Insecta | Diptera
SEQUEL | PBBCB286-17
Paratanytarsus | RRMFD762-15 | Insecta | Diptera
SEQUEL | PBBCB077-17
Micropsectra | RRMFA010-15 | Insecta | Diptera
SEQUEL | PBBCB134-17
Micropsectra | RRMFA124-15 | Insecta | Diptera
SEQUEL | PBBCB757-17
Micropsectra subletteorum | RRMFC1969-15 | Insecta | Diptera
SEQUEL | PBBCB234-17
Tanytarsus | RRMFD338-15 | Insecta | Diptera
SEQUEL | PBBCB603-17
Tanytarsus | RRMFI365-15 | Insecta | Diptera
SEQUEL | PBBCB648-17
Cladotanytarsus | RRMFE330-15 | Insecta | Diptera
SEQUEL | PBBCB090-17
Tanytarsus guerlus | RRMPC040-15 | Insecta | Diptera
SEQUEL | PBBCB786-17
Paratanytarsus | RRMPC020-15 | Insecta | Diptera
SEQUEL | PBBCB623-17
Dicrotendipes | RRMFC1614-15 | Insecta | Diptera
SEQUEL | PBBCB605-17
Chironominae | RRMFC1609-15 | Insecta | Diptera
SEQUEL | PBBCB337-17
Dicrotendipes tritomus | RRMPC1238-15 | Insecta | Diptera
SEQUEL | PBBCB824-17
Chironomus acidophilus | RRMFA058-15 | Insecta | Diptera
SEQUEL | PBBCB745-17
Chironomus melanescens | RRMFA118-15 | Insecta | Diptera

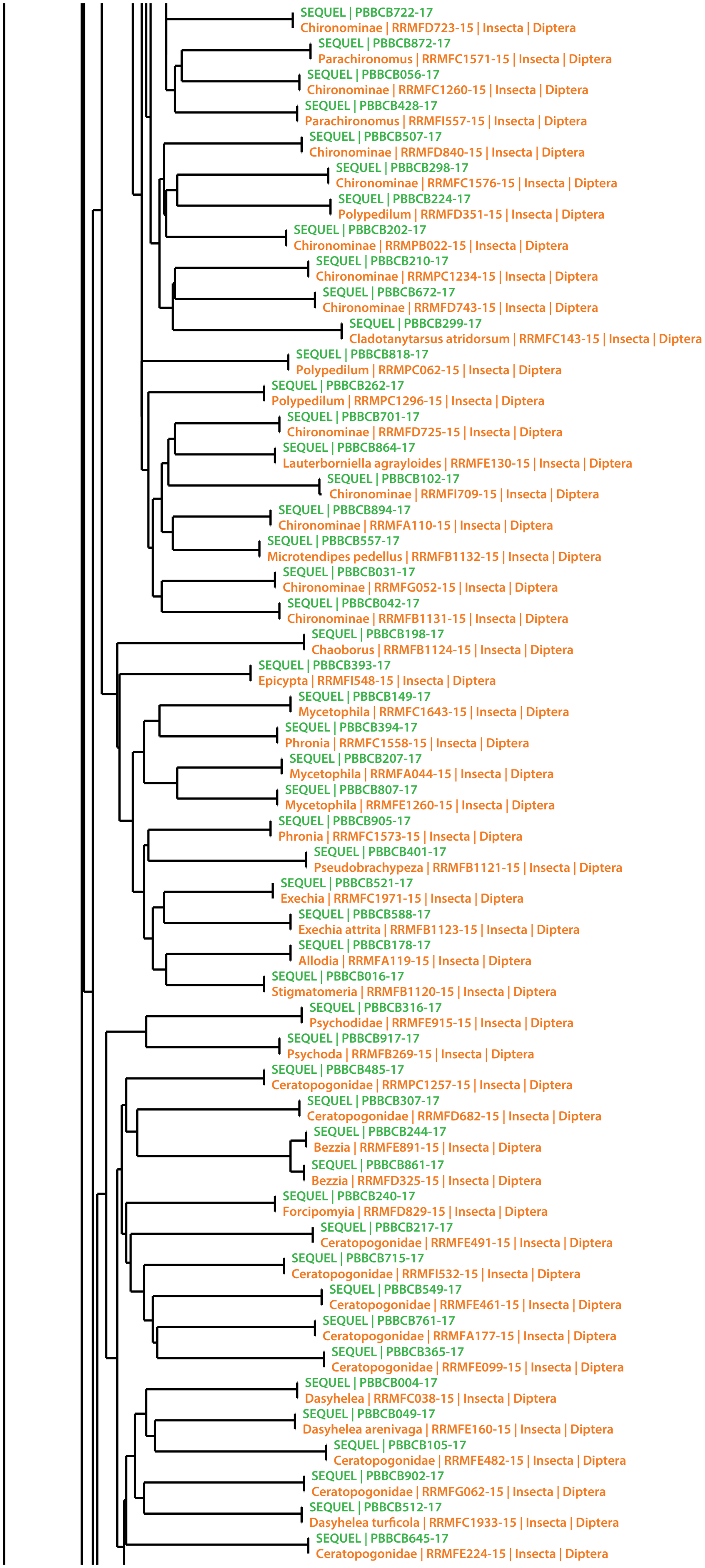
SEQUEL | PBBCB722-17
Chironominae | RRMFD723-15 | Insecta | Diptera
SEQUEL | PBBCB872-17
Parachironomus | RRMFC1571-15 | Insecta | Diptera
SEQUEL | PBBCB056-17
Chironominae | RRMFC1260-15 | Insecta | Diptera
SEQUEL | PBBCB428-17
Parachironomus | RRMFI557-15 | Insecta | Diptera
SEQUEL | PBBCB507-17
Chironominae | RRMFD840-15 | Insecta | Diptera
SEQUEL | PBBCB298-17
Chironominae | RRMFC1576-15 | Insecta | Diptera
SEQUEL | PBBCB224-17
Polypedilum | RRMFD351-15 | Insecta | Diptera
SEQUEL | PBBCB202-17
Chironominae | RRMPB022-15 | Insecta | Diptera
SEQUEL | PBBCB210-17
Chironominae | RRMPC1234-15 | Insecta | Diptera
SEQUEL | PBBCB672-17
Chironominae | RRMFD743-15 | Insecta | Diptera
SEQUEL | PBBCB299-17
Cladotanytarsus atridorsum | RRMFC143-15 | Insecta | Diptera
SEQUEL | PBBCB818-17
Polypedilum | RRMPC062-15 | Insecta | Diptera
SEQUEL | PBBCB262-17
Polypedilum | RRMPC1296-15 | Insecta | Diptera
SEQUEL | PBBCB701-17
Chironominae | RRMFD725-15 | Insecta | Diptera
SEQUEL | PBBCB864-17
Lauterborniella agrayloides | RRMFE130-15 | Insecta | Diptera
SEQUEL | PBBCB102-17
Chironominae | RRMFI709-15 | Insecta | Diptera
SEQUEL | PBBCB894-17
Chironominae | RRMFA110-15 | Insecta | Diptera
SEQUEL | PBBCB557-17
Microtendipes pedellus | RRMFB1132-15 | Insecta | Diptera
SEQUEL | PBBCB031-17
Chironominae | RRMFG052-15 | Insecta | Diptera
SEQUEL | PBBCB042-17
Chironominae | RRMFB1131-15 | Insecta | Diptera
SEQUEL | PBBCB198-17
Chaoborus | RRMFB1124-15 | Insecta | Diptera
SEQUEL | PBBCB393-17
Epicypta | RRMFI548-15 | Insecta | Diptera
SEQUEL | PBBCB149-17
Mycetophila | RRMFC1643-15 | Insecta | Diptera
SEQUEL | PBBCB394-17
Phronia | RRMFC1558-15 | Insecta | Diptera
SEQUEL | PBBCB207-17
Mycetophila | RRMFA044-15 | Insecta | Diptera
SEQUEL | PBBCB807-17
Mycetophila | RRMFE1260-15 | Insecta | Diptera
SEQUEL | PBBCB905-17
Phronia | RRMFC1573-15 | Insecta | Diptera
SEQUEL | PBBCB401-17
Pseudobrachypeza | RRMFB1121-15 | Insecta | Diptera
SEQUEL | PBBCB521-17
Exechia | RRMFC1971-15 | Insecta | Diptera
SEQUEL | PBBCB588-17
Exechia attrita | RRMFB1123-15 | Insecta | Diptera
SEQUEL | PBBCB178-17
Allodia | RRMFA119-15 | Insecta | Diptera
SEQUEL | PBBCB016-17
Stigmatomeria | RRMFB1120-15 | Insecta | Diptera
SEQUEL | PBBCB316-17
Psychodidae | RRMFE915-15 | Insecta | Diptera
SEQUEL | PBBCB917-17
Psychoda | RRMFB269-15 | Insecta | Diptera
SEQUEL | PBBCB485-17
Ceratopogonidae | RRMPC1257-15 | Insecta | Diptera
SEQUEL | PBBCB307-17
Ceratopogonidae | RRMFD682-15 | Insecta | Diptera
SEQUEL | PBBCB244-17
Bezzia | RRMFE891-15 | Insecta | Diptera
SEQUEL | PBBCB861-17
Bezzia | RRMFD325-15 | Insecta | Diptera
SEQUEL | PBBCB240-17
Forcipomyia | RRMFD829-15 | Insecta | Diptera
SEQUEL | PBBCB217-17
Ceratopogonidae | RRMFE491-15 | Insecta | Diptera
SEQUEL | PBBCB715-17
Ceratopogonidae | RRMFI532-15 | Insecta | Diptera
SEQUEL | PBBCB549-17
Ceratopogonidae | RRMFE461-15 | Insecta | Diptera
SEQUEL | PBBCB761-17
Ceratopogonidae | RRMFA177-15 | Insecta | Diptera
SEQUEL | PBBCB365-17
Ceratopogonidae | RRMFE099-15 | Insecta | Diptera
SEQUEL | PBBCB004-17
Dasyhelea | RRMFC038-15 | Insecta | Diptera
SEQUEL | PBBCB049-17
Dasyhelea arenivaga | RRMFE160-15 | Insecta | Diptera
SEQUEL | PBBCB105-17
Ceratopogonidae | RRMFE482-15 | Insecta | Diptera
SEQUEL | PBBCB902-17
Ceratopogonidae | RRMFG062-15 | Insecta | Diptera
SEQUEL | PBBCB512-17
Dasyhelea turficola | RRMFC1933-15 | Insecta | Diptera
SEQUEL | PBBCB645-17
Ceratopogonidae | RRMFE224-15 | Insecta | Diptera

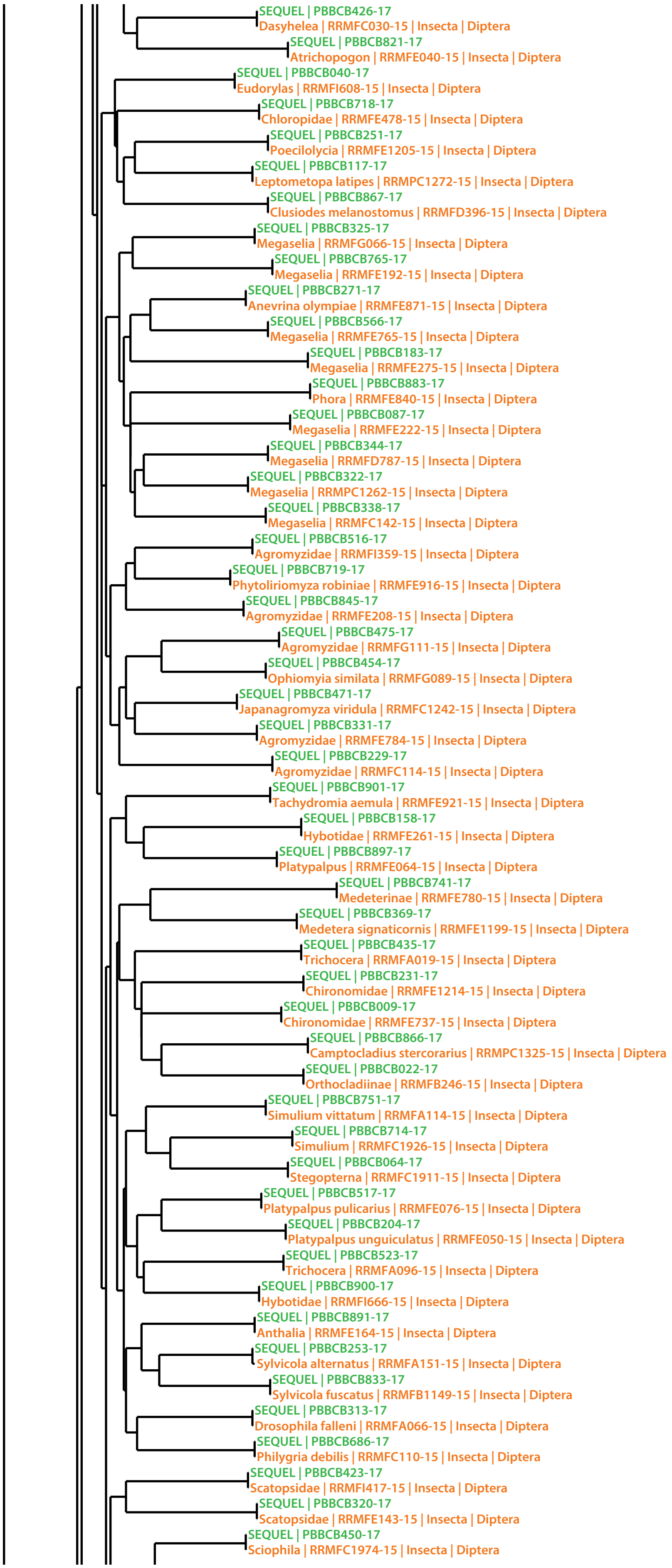
SEQUEL | PBBCB426-17
Dasyhelea | RRMFC030-15 | Insecta | Diptera
SEQUEL | PBBCB821-17
Atrichopogon | RRMFE040-15 | Insecta | Diptera
SEQUEL | PBBCB040-17
Eudorylas | RRMFI608-15 | Insecta | Diptera
SEQUEL | PBBCB718-17
Chloropidae | RRMFE478-15 | Insecta | Diptera
SEQUEL | PBBCB251-17
Poecilolycia | RRMFE1205-15 | Insecta | Diptera
SEQUEL | PBBCB117-17
Leptometopa latipes | RRMPC1272-15 | Insecta | Diptera
SEQUEL | PBBCB867-17
Clusiodes melanostomus | RRMFD396-15 | Insecta | Diptera
SEQUEL | PBBCB325-17
Megaselia | RRMFG066-15 | Insecta | Diptera
SEQUEL | PBBCB765-17
Megaselia | RRMFE192-15 | Insecta | Diptera
SEQUEL | PBBCB271-17
Anevrina olympiae | RRMFE871-15 | Insecta | Diptera
SEQUEL | PBBCB566-17
Megaselia | RRMFE765-15 | Insecta | Diptera
SEQUEL | PBBCB183-17
Megaselia | RRMFE275-15 | Insecta | Diptera
SEQUEL | PBBCB883-17
Phora | RRMFE840-15 | Insecta | Diptera
SEQUEL | PBBCB087-17
Megaselia | RRMFE222-15 | Insecta | Diptera
SEQUEL | PBBCB344-17
Megaselia | RRMFD787-15 | Insecta | Diptera
SEQUEL | PBBCB322-17
Megaselia | RRMPC1262-15 | Insecta | Diptera
SEQUEL | PBBCB338-17
Megaselia | RRMFC142-15 | Insecta | Diptera
SEQUEL | PBBCB516-17
Agromyzidae | RRMFI359-15 | Insecta | Diptera
SEQUEL | PBBCB719-17
Phytoliriomyza robiniae | RRMFE916-15 | Insecta | Diptera
SEQUEL | PBBCB845-17
Agromyzidae | RRMFE208-15 | Insecta | Diptera
SEQUEL | PBBCB475-17
Agromyzidae | RRMFG111-15 | Insecta | Diptera
SEQUEL | PBBCB454-17
Ophiomyia similata | RRMFG089-15 | Insecta | Diptera
SEQUEL | PBBCB471-17
Japanagromyza viridula | RRMFC1242-15 | Insecta | Diptera
SEQUEL | PBBCB331-17
Agromyzidae | RRMFE784-15 | Insecta | Diptera
SEQUEL | PBBCB229-17
Agromyzidae | RRMFC114-15 | Insecta | Diptera
SEQUEL | PBBCB901-17
Tachydromia aemula | RRMFE921-15 | Insecta | Diptera
SEQUEL | PBBCB158-17
Hybotidae | RRMFE261-15 | Insecta | Diptera
SEQUEL | PBBCB897-17
Platypalpus | RRMFE064-15 | Insecta | Diptera
SEQUEL | PBBCB741-17
Medeterinae | RRMFE780-15 | Insecta | Diptera
SEQUEL | PBBCB369-17
Medetera signaticornis | RRMFE1199-15 | Insecta | Diptera
SEQUEL | PBBCB435-17
Trichocera | RRMFA019-15 | Insecta | Diptera
SEQUEL | PBBCB231-17
Chironomidae | RRMFE1214-15 | Insecta | Diptera
SEQUEL | PBBCB009-17
Chironomidae | RRMFE737-15 | Insecta | Diptera
SEQUEL | PBBCB866-17
Camptocladius stercorarius | RRMPC1325-15 | Insecta | Diptera
SEQUEL | PBBCB022-17
Orthocladiinae | RRMFB246-15 | Insecta | Diptera
SEQUEL | PBBCB751-17
Simulium vittatum | RRMFA114-15 | Insecta | Diptera
SEQUEL | PBBCB714-17
Simulium | RRMFC1926-15 | Insecta | Diptera
SEQUEL | PBBCB064-17
Stegopterna | RRMFC1911-15 | Insecta | Diptera
SEQUEL | PBBCB517-17
Platypalpus pulicarius | RRMFE076-15 | Insecta | Diptera
SEQUEL | PBBCB204-17
Platypalpus unguiculatus | RRMFE050-15 | Insecta | Diptera
SEQUEL | PBBCB523-17
Trichocera | RRMFA096-15 | Insecta | Diptera
SEQUEL | PBBCB900-17
Hybotidae | RRMFI666-15 | Insecta | Diptera
SEQUEL | PBBCB891-17
Anthalia | RRMFE164-15 | Insecta | Diptera
SEQUEL | PBBCB253-17
Sylvicola alternatus | RRMFA151-15 | Insecta | Diptera
SEQUEL | PBBCB833-17
Sylvicola fuscatus | RRMFB1149-15 | Insecta | Diptera
SEQUEL | PBBCB313-17
Drosophila falleni | RRMFA066-15 | Insecta | Diptera
SEQUEL | PBBCB686-17
Philygria debilis | RRMFC110-15 | Insecta | Diptera
SEQUEL | PBBCB423-17
Scatopsidae | RRMFI417-15 | Insecta | Diptera
SEQUEL | PBBCB320-17
Scatopsidae | RRMFE143-15 | Insecta | Diptera
SEQUEL | PBBCB450-17
Sciophila | RRMFC1974-15 | Insecta | Diptera

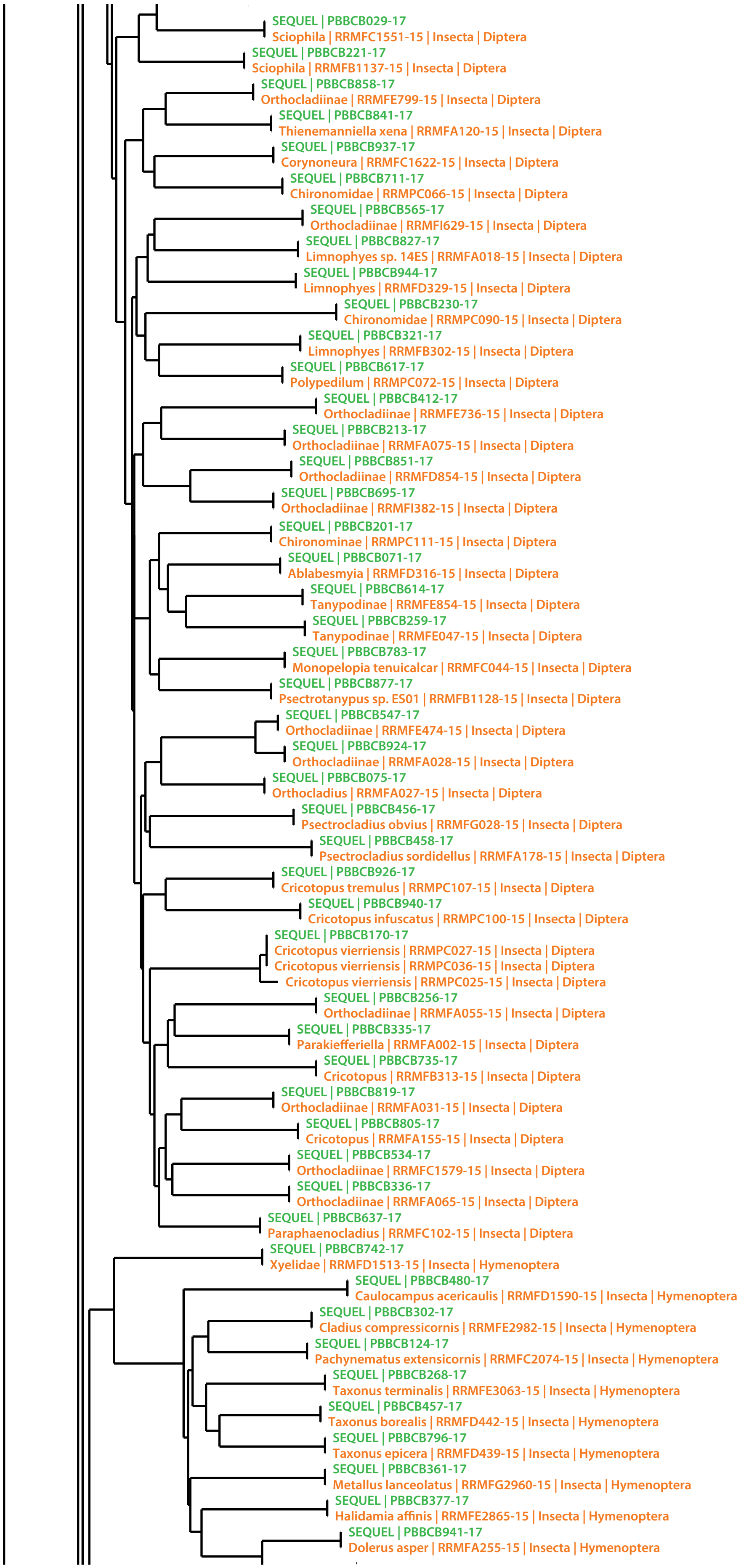
SEQUEL | PBBCB029-17
Sciophila | RRMFC1551-15 | Insecta | Diptera
SEQUEL | PBBCB221-17
Sciophila | RRMFB1137-15 | Insecta | Diptera
SEQUEL | PBBCB858-17
Orthocladiinae | RRMFE799-15 | Insecta | Diptera
SEQUEL | PBBCB841-17
Thienemanniella xena | RRMFA120-15 | Insecta | Diptera
SEQUEL | PBBCB937-17
Corynoneura | RRMFC1622-15 | Insecta | Diptera
SEQUEL | PBBCB711-17
Chironomidae | RRMPC066-15 | Insecta | Diptera
SEQUEL | PBBCB565-17
Orthocladiinae | RRMFI629-15 | Insecta | Diptera
SEQUEL | PBBCB827-17
Limnophyes sp. 14ES | RRMFA018-15 | Insecta | Diptera
SEQUEL | PBBCB944-17
Limnophyes | RRMFD329-15 | Insecta | Diptera
SEQUEL | PBBCB230-17
Chironomidae | RRMPC090-15 | Insecta | Diptera
SEQUEL | PBBCB321-17
Limnophyes | RRMFB302-15 | Insecta | Diptera
SEQUEL | PBBCB617-17
Polypedilum | RRMPC072-15 | Insecta | Diptera
SEQUEL | PBBCB412-17
Orthocladiinae | RRMFE736-15 | Insecta | Diptera
SEQUEL | PBBCB213-17
Orthocladiinae | RRMFA075-15 | Insecta | Diptera
SEQUEL | PBBCB851-17
Orthocladiinae | RRMFD854-15 | Insecta | Diptera
SEQUEL | PBBCB695-17
Orthocladiinae | RRMFI382-15 | Insecta | Diptera
SEQUEL | PBBCB201-17
Chironominae | RRMPC111-15 | Insecta | Diptera
SEQUEL | PBBCB071-17
Ablabesmyia | RRMFD316-15 | Insecta | Diptera
SEQUEL | PBBCB614-17
Tanypodinae | RRMFE854-15 | Insecta | Diptera
SEQUEL | PBBCB259-17
Tanypodinae | RRMFE047-15 | Insecta | Diptera
SEQUEL | PBBCB783-17
Monopelopia tenuicalcar | RRMFC044-15 | Insecta | Diptera
SEQUEL | PBBCB877-17
Psectrotanypus sp. ES01 | RRMFB1128-15 | Insecta | Diptera
SEQUEL | PBBCB547-17
Orthocladiinae | RRMFE474-15 | Insecta | Diptera
SEQUEL | PBBCB924-17
Orthocladiinae | RRMFA028-15 | Insecta | Diptera
SEQUEL | PBBCB075-17
Orthocladius | RRMFA027-15 | Insecta | Diptera
SEQUEL | PBBCB456-17
Psectrocladius obvius | RRMFG028-15 | Insecta | Diptera
SEQUEL | PBBCB458-17
Psectrocladius sordidellus | RRMFA178-15 | Insecta | Diptera
SEQUEL | PBBCB926-17
Cricotopus tremulus | RRMPC107-15 | Insecta | Diptera
SEQUEL | PBBCB940-17
Cricotopus infuscatus | RRMPC100-15 | Insecta | Diptera
SEQUEL | PBBCB170-17
Cricotopus vierriensis | RRMPC027-15 | Insecta | Diptera
Cricotopus vierriensis | RRMPC036-15 | Insecta | Diptera
Cricotopus vierriensis | RRMPC025-15 | Insecta | Diptera
SEQUEL | PBBCB256-17
Orthocladiinae | RRMFA055-15 | Insecta | Diptera
SEQUEL | PBBCB335-17
Parakiefferiella | RRMFA002-15 | Insecta | Diptera
SEQUEL | PBBCB735-17
Cricotopus | RRMFB313-15 | Insecta | Diptera
SEQUEL | PBBCB819-17
Orthocladiinae | RRMFA031-15 | Insecta | Diptera
SEQUEL | PBBCB805-17
Cricotopus | RRMFA155-15 | Insecta | Diptera
SEQUEL | PBBCB534-17
Orthocladiinae | RRMFC1579-15 | Insecta | Diptera
SEQUEL | PBBCB336-17
Orthocladiinae | RRMFA065-15 | Insecta | Diptera
SEQUEL | PBBCB637-17
Paraphaenocladius | RRMFC102-15 | Insecta | Diptera
SEQUEL | PBBCB742-17
Xyelidae | RRMFD1513-15 | Insecta | Hymenoptera
SEQUEL | PBBCB480-17
Caulocampus acericaulis | RRMFD1590-15 | Insecta | Hymenoptera
SEQUEL | PBBCB302-17
Cladius compressicornis | RRMFE2982-15 | Insecta | Hymenoptera
SEQUEL | PBBCB124-17
Pachynematus extensicornis | RRMFC2074-15 | Insecta | Hymenoptera
SEQUEL | PBBCB268-17
Taxonus terminalis | RRMFE3063-15 | Insecta | Hymenoptera
SEQUEL | PBBCB457-17
Taxonus borealis | RRMFD442-15 | Insecta | Hymenoptera
SEQUEL | PBBCB796-17
Taxonus epicera | RRMFD439-15 | Insecta | Hymenoptera
SEQUEL | PBBCB361-17
Metallus lanceolatus | RRMFG2960-15 | Insecta | Hymenoptera
SEQUEL | PBBCB377-17
Halidamia affinis | RRMFE2865-15 | Insecta | Hymenoptera
SEQUEL | PBBCB941-17
Dolerus asper | RRMFA255-15 | Insecta | Hymenoptera

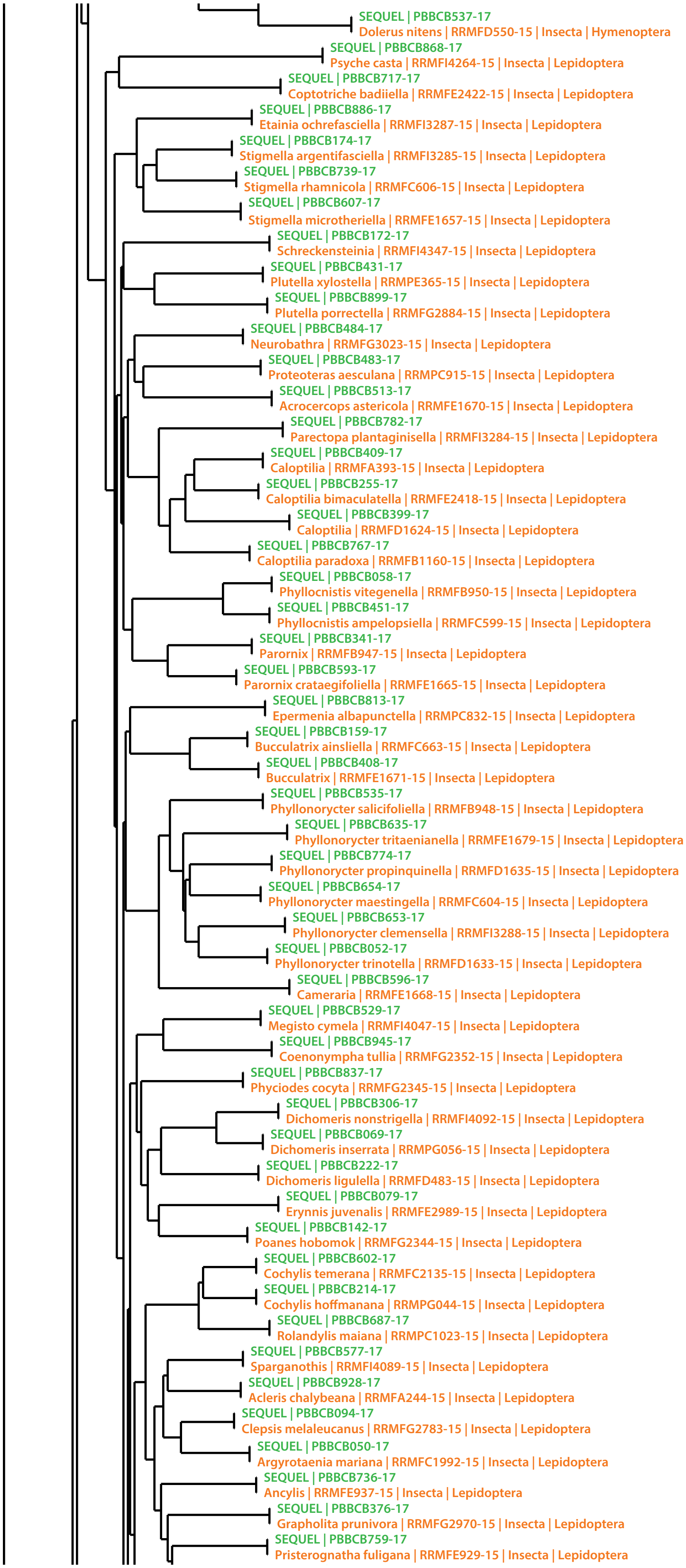
SEQUEL | PBBCB537-17
Dolerus nitens | RRMFD550-15 | Insecta | Hymenoptera
SEQUEL | PBBCB868-17
Psyche casta | RRMFI4264-15 | Insecta | Lepidoptera
SEQUEL | PBBCB717-17
Coptotriche badiiella | RRMFE2422-15 | Insecta | Lepidoptera
SEQUEL | PBBCB886-17
Etainia ochrefasciella | RRMFI3287-15 | Insecta | Lepidoptera
SEQUEL | PBBCB174-17
Stigmella argentifasciella | RRMFI3285-15 | Insecta | Lepidoptera
SEQUEL | PBBCB739-17
Stigmella rhamnicola | RRMFC606-15 | Insecta | Lepidoptera
SEQUEL | PBBCB607-17
Stigmella microtheriella | RRMFE1657-15 | Insecta | Lepidoptera
SEQUEL | PBBCB172-17
Schreckensteinia | RRMFI4347-15 | Insecta | Lepidoptera
SEQUEL | PBBCB431-17
Plutella xylostella | RRMPE365-15 | Insecta | Lepidoptera
SEQUEL | PBBCB899-17
Plutella porrectella | RRMFG2884-15 | Insecta | Lepidoptera
SEQUEL | PBBCB484-17
Neurobathra | RRMFG3023-15 | Insecta | Lepidoptera
SEQUEL | PBBCB483-17
Proteoteras aesculana | RRMPC915-15 | Insecta | Lepidoptera
SEQUEL | PBBCB513-17
Acrocercops astericola | RRMFE1670-15 | Insecta | Lepidoptera
SEQUEL | PBBCB782-17
Parectopa plantaginisella | RRMFI3284-15 | Insecta | Lepidoptera
SEQUEL | PBBCB409-17
Caloptilia | RRMFA393-15 | Insecta | Lepidoptera
SEQUEL | PBBCB255-17
Caloptilia bimaculatella | RRMFE2418-15 | Insecta | Lepidoptera
SEQUEL | PBBCB399-17
Caloptilia | RRMFD1624-15 | Insecta | Lepidoptera
SEQUEL | PBBCB767-17
Caloptilia paradoxa | RRMFB1160-15 | Insecta | Lepidoptera
SEQUEL | PBBCB058-17
Phyllocnistis vitegenella | RRMFB950-15 | Insecta | Lepidoptera
SEQUEL | PBBCB451-17
Phyllocnistis ampelopsiella | RRMFC599-15 | Insecta | Lepidoptera
SEQUEL | PBBCB341-17
Parornix | RRMFB947-15 | Insecta | Lepidoptera
SEQUEL | PBBCB593-17
Parornix crataegifoliella | RRMFE1665-15 | Insecta | Lepidoptera
SEQUEL | PBBCB813-17
Epermenia albapunctella | RRMPC832-15 | Insecta | Lepidoptera
SEQUEL | PBBCB159-17
Bucculatrix ainsliella | RRMFC663-15 | Insecta | Lepidoptera
SEQUEL | PBBCB408-17
Bucculatrix | RRMFE1671-15 | Insecta | Lepidoptera
SEQUEL | PBBCB535-17
Phyllonorycter salicifoliella | RRMFB948-15 | Insecta | Lepidoptera
SEQUEL | PBBCB635-17
Phyllonorycter tritaenianella | RRMFE1679-15 | Insecta | Lepidoptera
SEQUEL | PBBCB774-17
Phyllonorycter propinquinella | RRMFD1635-15 | Insecta | Lepidoptera
SEQUEL | PBBCB654-17
Phyllonorycter maestingella | RRMFC604-15 | Insecta | Lepidoptera
SEQUEL | PBBCB653-17
Phyllonorycter clemensella | RRMFI3288-15 | Insecta | Lepidoptera
SEQUEL | PBBCB052-17
Phyllonorycter trinotella | RRMFD1633-15 | Insecta | Lepidoptera
SEQUEL | PBBCB596-17
Cameraria | RRMFE1668-15 | Insecta | Lepidoptera
SEQUEL | PBBCB529-17
Megisto cymela | RRMFI4047-15 | Insecta | Lepidoptera
SEQUEL | PBBCB945-17
Coenonympha tullia | RRMFG2352-15 | Insecta | Lepidoptera
SEQUEL | PBBCB837-17
Phyciodes cocyta | RRMFG2345-15 | Insecta | Lepidoptera
SEQUEL | PBBCB306-17
Dichomeris nonstrigella | RRMFI4092-15 | Insecta | Lepidoptera
SEQUEL | PBBCB069-17
Dichomeris inserrata | RRMPG056-15 | Insecta | Lepidoptera
SEQUEL | PBBCB222-17
Dichomeris ligulella | RRMFD483-15 | Insecta | Lepidoptera
SEQUEL | PBBCB079-17
Erynnis juvenalis | RRMFE2989-15 | Insecta | Lepidoptera
SEQUEL | PBBCB142-17
Poanes hobomok | RRMFG2344-15 | Insecta | Lepidoptera
SEQUEL | PBBCB602-17
Cochylis temerana | RRMFC2135-15 | Insecta | Lepidoptera
SEQUEL | PBBCB214-17
Cochylis hoffmanana | RRMPG044-15 | Insecta | Lepidoptera
SEQUEL | PBBCB687-17
Rolandylis maiana | RRMPC1023-15 | Insecta | Lepidoptera
SEQUEL | PBBCB577-17
Sparganothis | RRMFI4089-15 | Insecta | Lepidoptera
SEQUEL | PBBCB928-17
Acleris chalybeana | RRMFA244-15 | Insecta | Lepidoptera
SEQUEL | PBBCB094-17
Clepsis melaleucanus | RRMFG2783-15 | Insecta | Lepidoptera
SEQUEL | PBBCB050-17
Argyrotaenia mariana | RRMFC1992-15 | Insecta | Lepidoptera
SEQUEL | PBBCB736-17
Ancylis | RRMFE937-15 | Insecta | Lepidoptera
SEQUEL | PBBCB376-17
Grapholita prunivora | RRMFG2970-15 | Insecta | Lepidoptera
SEQUEL | PBBCB759-17
Pristerognatha fuligana | RRMFE929-15 | Insecta | Lepidoptera

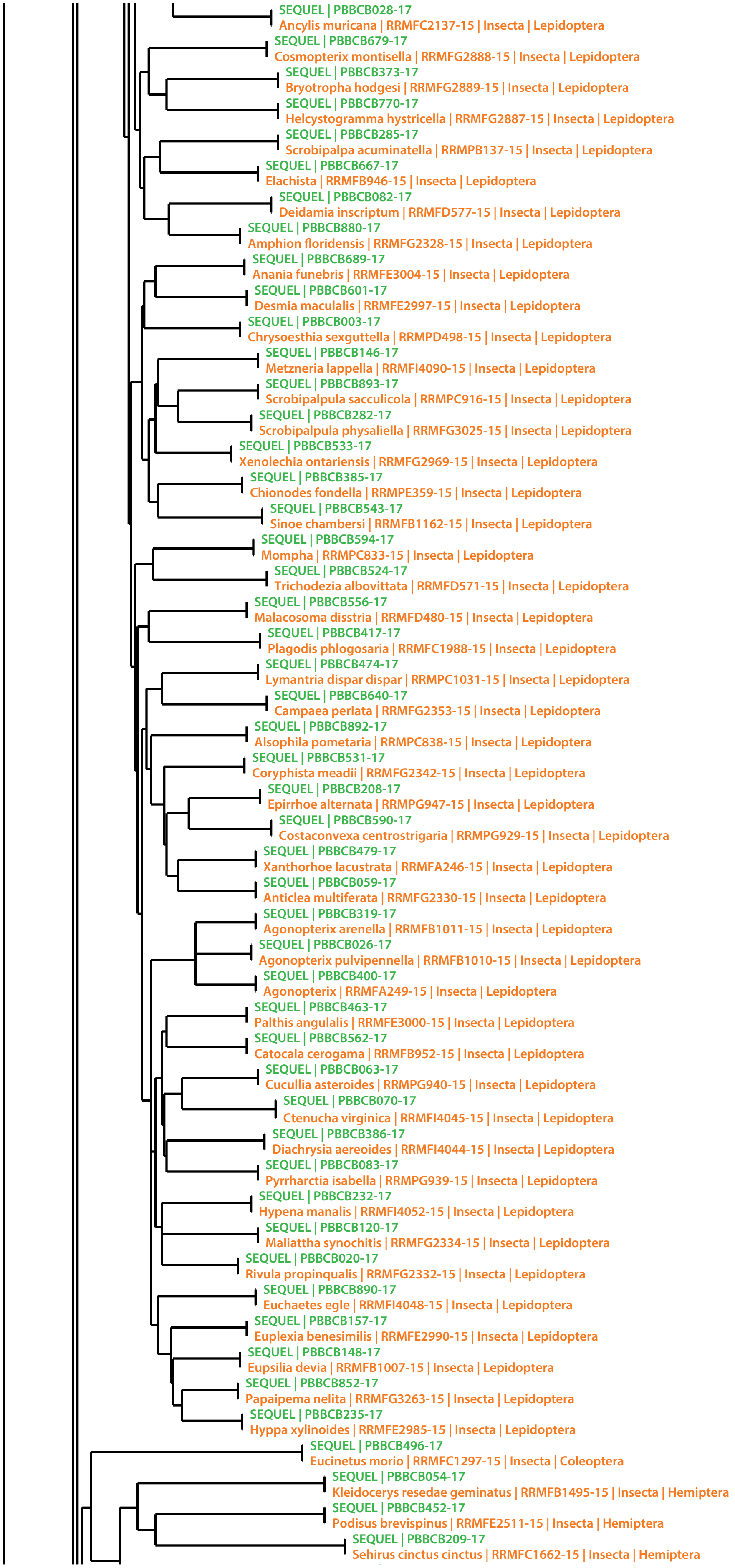
SEQUEL | PBBCB028-17
Ancylis muricana | RRMFC2137-15 | Insecta | Lepidoptera
SEQUEL | PBBCB679-17
Cosmopterix montisella | RRMFG2888-15 | Insecta | Lepidoptera
SEQUEL | PBBCB373-17
Bryotropha hodgesi | RRMFG2889-15 | Insecta | Lepidoptera
SEQUEL | PBBCB770-17
Helcystogramma hystricella | RRMFG2887-15 | Insecta | Lepidoptera
SEQUEL | PBBCB285-17
Scrobipalpa acuminatella | RRMPB137-15 | Insecta | Lepidoptera
SEQUEL | PBBCB667-17
Elachista | RRMFB946-15 | Insecta | Lepidoptera
SEQUEL | PBBCB082-17
Deidamia inscriptum | RRMFD577-15 | Insecta | Lepidoptera
SEQUEL | PBBCB880-17
Amphion floridensis | RRMFG2328-15 | Insecta | Lepidoptera
SEQUEL | PBBCB689-17
Anania funebris | RRMFE3004-15 | Insecta | Lepidoptera
SEQUEL | PBBCB601-17
Desmia maculalis | RRMFE2997-15 | Insecta | Lepidoptera
SEQUEL | PBBCB003-17
Chrysoesthia sexguttella | RRMPD498-15 | Insecta | Lepidoptera
SEQUEL | PBBCB146-17
Metzneria lappella | RRMFI4090-15 | Insecta | Lepidoptera
SEQUEL | PBBCB893-17
Scrobipalpula sacculicola | RRMPC916-15 | Insecta | Lepidoptera
SEQUEL | PBBCB282-17
Scrobipalpula physaliella | RRMFG3025-15 | Insecta | Lepidoptera
SEQUEL | PBBCB533-17
Xenolechia ontariensis | RRMFG2969-15 | Insecta | Lepidoptera
SEQUEL | PBBCB385-17
Chionodes fondella | RRMPE359-15 | Insecta | Lepidoptera
SEQUEL | PBBCB543-17
Sinoe chambersi | RRMFB1162-15 | Insecta | Lepidoptera
SEQUEL | PBBCB594-17
Mompha | RRMPC833-15 | Insecta | Lepidoptera
SEQUEL | PBBCB524-17
Trichodezia albovittata | RRMFD571-15 | Insecta | Lepidoptera
SEQUEL | PBBCB556-17
Malacosoma disstria | RRMFD480-15 | Insecta | Lepidoptera
SEQUEL | PBBCB417-17
Plagodis phlogosaria | RRMFC1988-15 | Insecta | Lepidoptera
SEQUEL | PBBCB474-17
Lymantria dispar dispar | RRMPC1031-15 | Insecta | Lepidoptera
SEQUEL | PBBCB640-17
Campaea perlata | RRMFG2353-15 | Insecta | Lepidoptera
SEQUEL | PBBCB892-17
Alsophila pometaria | RRMPC838-15 | Insecta | Lepidoptera
SEQUEL | PBBCB531-17
Coryphista meadii | RRMFG2342-15 | Insecta | Lepidoptera
SEQUEL | PBBCB208-17
Epirrhoe alternata | RRMPG947-15 | Insecta | Lepidoptera
SEQUEL | PBBCB590-17
Costaconvexa centrostrigaria | RRMPG929-15 | Insecta | Lepidoptera
SEQUEL | PBBCB479-17
Xanthorhoe lacustrata | RRMFA246-15 | Insecta | Lepidoptera
SEQUEL | PBBCB059-17
Anticlea multiferata | RRMFG2330-15 | Insecta | Lepidoptera
SEQUEL | PBBCB319-17
Agonopterix arenella | RRMFB1011-15 | Insecta | Lepidoptera
SEQUEL | PBBCB026-17
Agonopterix pulvipennella | RRMFB1010-15 | Insecta | Lepidoptera
SEQUEL | PBBCB400-17
Agonopterix | RRMFA249-15 | Insecta | Lepidoptera
SEQUEL | PBBCB463-17
Palthis angulalis | RRMFE3000-15 | Insecta | Lepidoptera
SEQUEL | PBBCB562-17
Catocala cerogama | RRMFB952-15 | Insecta | Lepidoptera
SEQUEL | PBBCB063-17
Cucullia asteroides | RRMPG940-15 | Insecta | Lepidoptera
SEQUEL | PBBCB070-17
Ctenucha virginica | RRMFI4045-15 | Insecta | Lepidoptera
SEQUEL | PBBCB386-17
Diachrysia aereoides | RRMFI4044-15 | Insecta | Lepidoptera
SEQUEL | PBBCB083-17
Pyrrharctia isabella | RRMPG939-15 | Insecta | Lepidoptera
SEQUEL | PBBCB232-17
Hypena manalis | RRMFI4052-15 | Insecta | Lepidoptera
SEQUEL | PBBCB120-17
Maliattha synochitis | RRMFG2334-15 | Insecta | Lepidoptera
SEQUEL | PBBCB020-17
Rivula propinqualis | RRMFG2332-15 | Insecta | Lepidoptera
SEQUEL | PBBCB890-17
Euchaetes egle | RRMFI4048-15 | Insecta | Lepidoptera
SEQUEL | PBBCB157-17
Euplexia benesimilis | RRMFE2990-15 | Insecta | Lepidoptera
SEQUEL | PBBCB148-17
Eupsilia devia | RRMFB1007-15 | Insecta | Lepidoptera
SEQUEL | PBBCB852-17
Papaipema nelita | RRMFG3263-15 | Insecta | Lepidoptera
SEQUEL | PBBCB235-17
Hyppa xylinoides | RRMFE2985-15 | Insecta | Lepidoptera
SEQUEL | PBBCB496-17
Eucinetus morio | RRMFC1297-15 | Insecta | Coleoptera
SEQUEL | PBBCB054-17
Kleidocerys resedae geminatus | RRMFB1495-15 | Insecta | Hemiptera
SEQUEL | PBBCB452-17
Podisus brevispinus | RRMFE2511-15 | Insecta | Hemiptera
SEQUEL | PBBCB209-17
Sehirus cinctus cinctus | RRMFC1662-15 | Insecta | Hemiptera

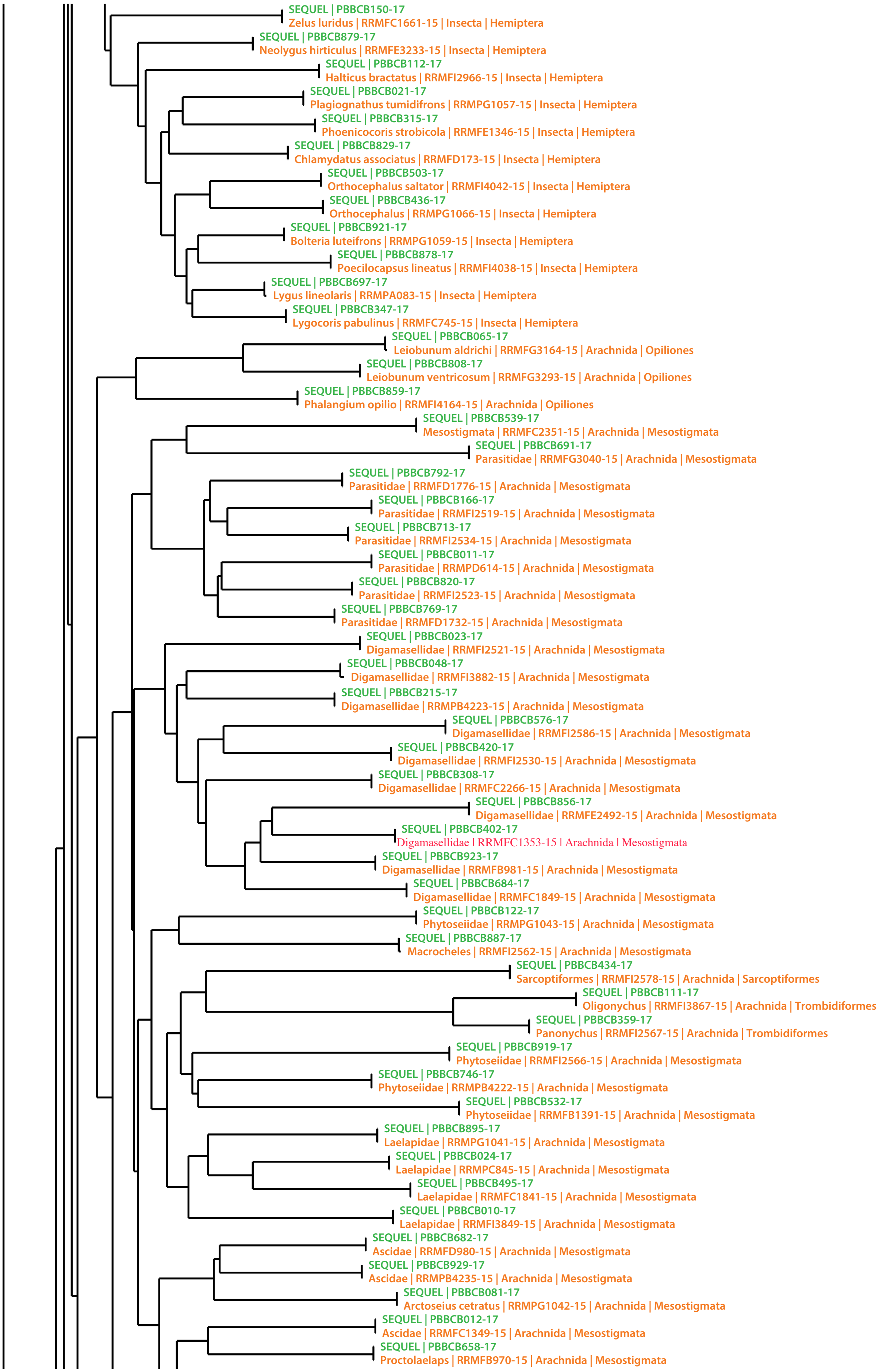
SEQUEL | PBBCB150-17
Zelus luridus | RRMFC1661-15 | Insecta | Hemiptera
SEQUEL | PBBCB879-17
Neolygus hirticulus | RRMFE3233-15 | Insecta | Hemiptera
SEQUEL | PBBCB112-17
Halticus bractatus | RRMFI2966-15 | Insecta | Hemiptera
SEQUEL | PBBCB021-17
Plagiognathus tumidifrons | RRMPG1057-15 | Insecta | Hemiptera
SEQUEL | PBBCB315-17
Phoenicocoris strobicola | RRMFE1346-15 | Insecta | Hemiptera
SEQUEL | PBBCB829-17
Chlamydatus associatus | RRMFD173-15 | Insecta | Hemiptera
SEQUEL | PBBCB503-17
Orthocephalus saltator | RRMFI4042-15 | Insecta | Hemiptera
SEQUEL | PBBCB436-17
Orthocephalus | RRMPG1066-15 | Insecta | Hemiptera
SEQUEL | PBBCB921-17
Bolteria luteifrons | RRMPG1059-15 | Insecta | Hemiptera
SEQUEL | PBBCB878-17
Poecilocapsus lineatus | RRMFI4038-15 | Insecta | Hemiptera
SEQUEL | PBBCB697-17
Lygus lineolaris | RRMPA083-15 | Insecta | Hemiptera
SEQUEL | PBBCB347-17
Lygocoris pabulinus | RRMFC745-15 | Insecta | Hemiptera
SEQUEL | PBBCB065-17
Leiobunum aldrichi | RRMFG3164-15 | Arachnida | Opiliones
SEQUEL | PBBCB808-17
Leiobunum ventricosum | RRMFG3293-15 | Arachnida | Opiliones
SEQUEL | PBBCB859-17
Phalangium opilio | RRMFI4164-15 | Arachnida | Opiliones
SEQUEL | PBBCB539-17
Mesostigmata | RRMFC2351-15 | Arachnida | Mesostigmata
SEQUEL | PBBCB691-17
Parasitidae | RRMFG3040-15 | Arachnida | Mesostigmata
SEQUEL | PBBCB792-17
Parasitidae | RRMFD1776-15 | Arachnida | Mesostigmata
SEQUEL | PBBCB166-17
Parasitidae | RRMFI2519-15 | Arachnida | Mesostigmata
SEQUEL | PBBCB713-17
Parasitidae | RRMFI2534-15 | Arachnida | Mesostigmata
SEQUEL | PBBCB011-17
Parasitidae | RRMPD614-15 | Arachnida | Mesostigmata
SEQUEL | PBBCB820-17
Parasitidae | RRMFI2523-15 | Arachnida | Mesostigmata
SEQUEL | PBBCB769-17
Parasitidae | RRMFD1732-15 | Arachnida | Mesostigmata
SEQUEL | PBBCB023-17
Digamasellidae | RRMFI2521-15 | Arachnida | Mesostigmata
SEQUEL | PBBCB048-17
Digamasellidae | RRMFI3882-15 | Arachnida | Mesostigmata
SEQUEL | PBBCB215-17
Digamasellidae | RRMPB4223-15 | Arachnida | Mesostigmata
SEQUEL | PBBCB576-17
Digamasellidae | RRMFI2586-15 | Arachnida | Mesostigmata
SEQUEL | PBBCB420-17
Digamasellidae | RRMFI2530-15 | Arachnida | Mesostigmata
SEQUEL | PBBCB308-17
Digamasellidae | RRMFC2266-15 | Arachnida | Mesostigmata
SEQUEL | PBBCB856-17
Digamasellidae | RRMFE2492-15 | Arachnida | Mesostigmata
SEQUEL | PBBCB402-17
Digamasellidae | RRMFC1353-15 | Arachnida | Mesostigmata
SEQUEL | PBBCB923-17
Digamasellidae | RRMFB981-15 | Arachnida | Mesostigmata
SEQUEL | PBBCB684-17
Digamasellidae | RRMFC1849-15 | Arachnida | Mesostigmata
SEQUEL | PBBCB122-17
Phytoseiidae | RRMPG1043-15 | Arachnida | Mesostigmata
SEQUEL | PBBCB887-17
Macrocheles | RRMFI2562-15 | Arachnida | Mesostigmata
SEQUEL | PBBCB434-17
Sarcoptiformes | RRMFI2578-15 | Arachnida | Sarcoptiformes
SEQUEL | PBBCB111-17
Oligonychus | RRMFI3867-15 | Arachnida | Trombidiformes
SEQUEL | PBBCB359-17
Panonychus | RRMFI2567-15 | Arachnida | Trombidiformes
SEQUEL | PBBCB919-17
Phytoseiidae | RRMFI2566-15 | Arachnida | Mesostigmata
SEQUEL | PBBCB746-17
Phytoseiidae | RRMPB4222-15 | Arachnida | Mesostigmata
SEQUEL | PBBCB532-17
Phytoseiidae | RRMFB1391-15 | Arachnida | Mesostigmata
SEQUEL | PBBCB895-17
Laelapidae | RRMPG1041-15 | Arachnida | Mesostigmata
SEQUEL | PBBCB024-17
Laelapidae | RRMPC845-15 | Arachnida | Mesostigmata
SEQUEL | PBBCB495-17
Laelapidae | RRMFC1841-15 | Arachnida | Mesostigmata
SEQUEL | PBBCB010-17
Laelapidae | RRMFI3849-15 | Arachnida | Mesostigmata
SEQUEL | PBBCB682-17
Ascidae | RRMFD980-15 | Arachnida | Mesostigmata
SEQUEL | PBBCB929-17
Ascidae | RRMPB4235-15 | Arachnida | Mesostigmata
SEQUEL | PBBCB081-17
Arctoseius cetratus | RRMPG1042-15 | Arachnida | Mesostigmata
SEQUEL | PBBCB012-17
Ascidae | RRMFC1349-15 | Arachnida | Mesostigmata
SEQUEL | PBBCB658-17
Proctolaelaps | RRMFB970-15 | Arachnida | Mesostigmata

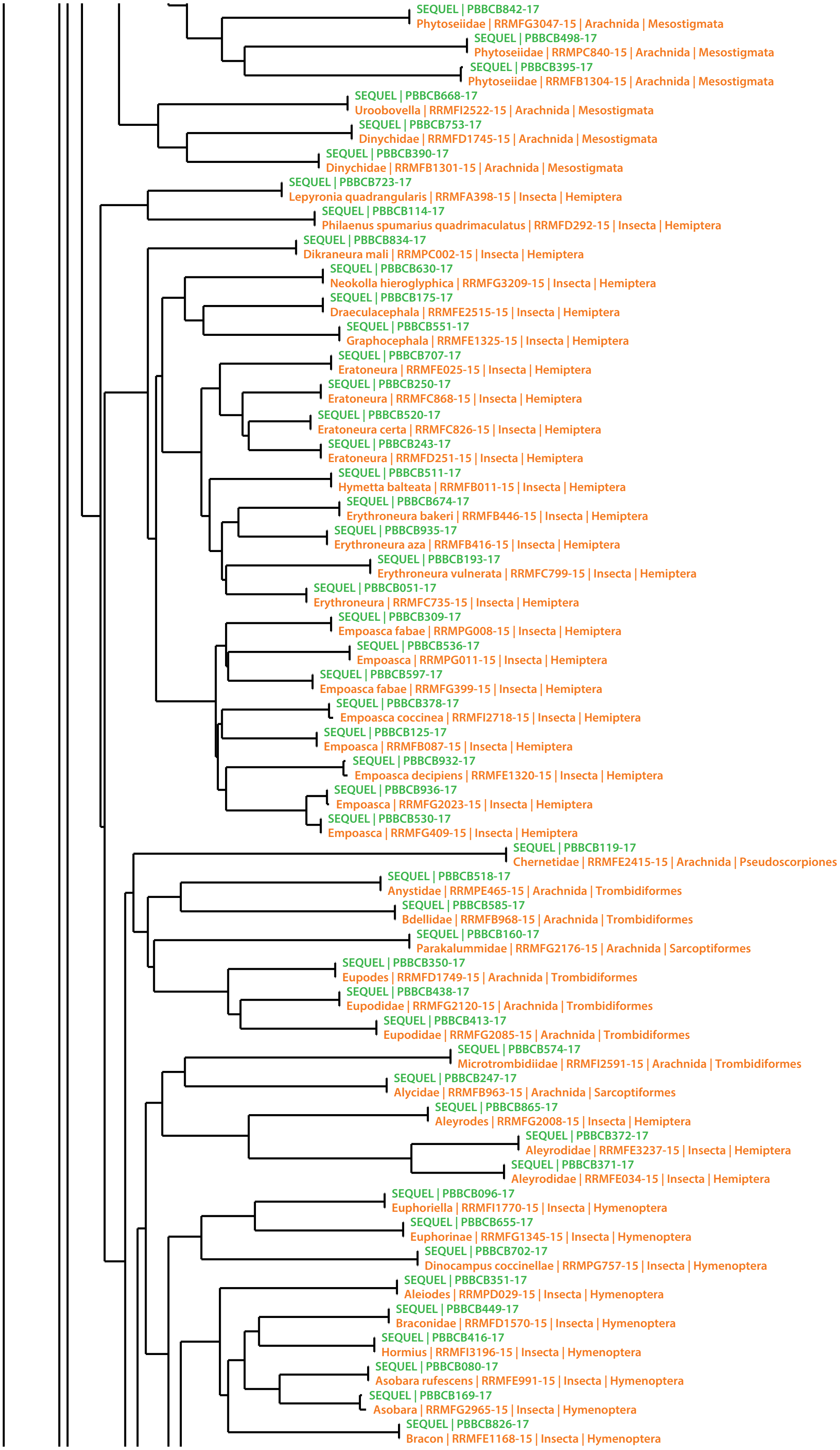
SEQUEL | PBBCB842-17
Phytoseiidae | RRMFG3047-15 | Arachnida | Mesostigmata
SEQUEL | PBBCB498-17
Phytoseiidae | RRMPC840-15 | Arachnida | Mesostigmata
SEQUEL | PBBCB395-17
Phytoseiidae | RRMFB1304-15 | Arachnida | Mesostigmata
SEQUEL | PBBCB668-17
Uroobovella | RRMFI2522-15 | Arachnida | Mesostigmata
SEQUEL | PBBCB753-17
Dinychidae | RRMFD1745-15 | Arachnida | Mesostigmata
SEQUEL | PBBCB390-17
Dinychidae | RRMFB1301-15 | Arachnida | Mesostigmata
SEQUEL | PBBCB723-17
Lepyronia quadrangularis | RRMFA398-15 | Insecta | Hemiptera
SEQUEL | PBBCB114-17
Philaenus spumarius quadrimaculatus | RRMFD292-15 | Insecta | Hemiptera
SEQUEL | PBBCB834-17
Dikraneura mali | RRMPC002-15 | Insecta | Hemiptera
SEQUEL | PBBCB630-17
Neokolla hieroglyphica | RRMFG3209-15 | Insecta | Hemiptera
SEQUEL | PBBCB175-17
Draeculacephala | RRMFE2515-15 | Insecta | Hemiptera
SEQUEL | PBBCB551-17
Graphocephala | RRMFE1325-15 | Insecta | Hemiptera
SEQUEL | PBBCB707-17
Eratoneura | RRMFE025-15 | Insecta | Hemiptera
SEQUEL | PBBCB250-17
Eratoneura | RRMFC868-15 | Insecta | Hemiptera
SEQUEL | PBBCB520-17
Eratoneura certa | RRMFC826-15 | Insecta | Hemiptera
SEQUEL | PBBCB243-17
Eratoneura | RRMFD251-15 | Insecta | Hemiptera
SEQUEL | PBBCB511-17
Hymetta balteata | RRMFB011-15 | Insecta | Hemiptera
SEQUEL | PBBCB674-17
Erythroneura bakeri | RRMFB446-15 | Insecta | Hemiptera
SEQUEL | PBBCB935-17
Erythroneura aza | RRMFB416-15 | Insecta | Hemiptera
SEQUEL | PBBCB193-17
Erythroneura vulnerata | RRMFC799-15 | Insecta | Hemiptera
SEQUEL | PBBCB051-17
Erythroneura | RRMFC735-15 | Insecta | Hemiptera
SEQUEL | PBBCB309-17
Empoasca fabae | RRMPG008-15 | Insecta | Hemiptera
SEQUEL | PBBCB536-17
Empoasca | RRMPG011-15 | Insecta | Hemiptera
SEQUEL | PBBCB597-17
Empoasca fabae | RRMFG399-15 | Insecta | Hemiptera
SEQUEL | PBBCB378-17
Empoasca coccinea | RRMFI2718-15 | Insecta | Hemiptera
SEQUEL | PBBCB125-17
Empoasca | RRMFB087-15 | Insecta | Hemiptera
SEQUEL | PBBCB932-17
Empoasca decipiens | RRMFE1320-15 | Insecta | Hemiptera
SEQUEL | PBBCB936-17
Empoasca | RRMFG2023-15 | Insecta | Hemiptera
SEQUEL | PBBCB530-17
Empoasca | RRMFG409-15 | Insecta | Hemiptera
SEQUEL | PBBCB119-17
Chernetidae | RRMFE2415-15 | Arachnida | Pseudoscorpiones
SEQUEL | PBBCB518-17
Anystidae | RRMPE465-15 | Arachnida | Trombidiformes
SEQUEL | PBBCB585-17
Bdellidae | RRMFB968-15 | Arachnida | Trombidiformes
SEQUEL | PBBCB160-17
Parakalummidae | RRMFG2176-15 | Arachnida | Sarcoptiformes
SEQUEL | PBBCB350-17
Eupodes | RRMFD1749-15 | Arachnida | Trombidiformes
SEQUEL | PBBCB438-17
Eupodidae | RRMFG2120-15 | Arachnida | Trombidiformes
SEQUEL | PBBCB413-17
Eupodidae | RRMFG2085-15 | Arachnida | Trombidiformes
SEQUEL | PBBCB574-17
Microtrombidiidae | RRMFI2591-15 | Arachnida | Trombidiformes
SEQUEL | PBBCB247-17
Alycidae | RRMFB963-15 | Arachnida | Sarcoptiformes
SEQUEL | PBBCB865-17
Aleyrodes | RRMFG2008-15 | Insecta | Hemiptera
SEQUEL | PBBCB372-17
Aleyrodidae | RRMFE3237-15 | Insecta | Hemiptera
SEQUEL | PBBCB371-17
Aleyrodidae | RRMFE034-15 | Insecta | Hemiptera
SEQUEL | PBBCB096-17
Euphoriella | RRMFI1770-15 | Insecta | Hymenoptera
SEQUEL | PBBCB655-17
Euphorinae | RRMFG1345-15 | Insecta | Hymenoptera
SEQUEL | PBBCB702-17
Dinocampus coccinellae | RRMPG757-15 | Insecta | Hymenoptera
SEQUEL | PBBCB351-17
Aleiodes | RRMPD029-15 | Insecta | Hymenoptera
SEQUEL | PBBCB449-17
Braconidae | RRMFD1570-15 | Insecta | Hymenoptera
SEQUEL | PBBCB416-17
Hormius | RRMFI3196-15 | Insecta | Hymenoptera
SEQUEL | PBBCB080-17
Asobara rufescens | RRMFE991-15 | Insecta | Hymenoptera
SEQUEL | PBBCB169-17
Asobara | RRMFG2965-15 | Insecta | Hymenoptera
SEQUEL | PBBCB826-17
Bracon | RRMFE1168-15 | Insecta | Hymenoptera

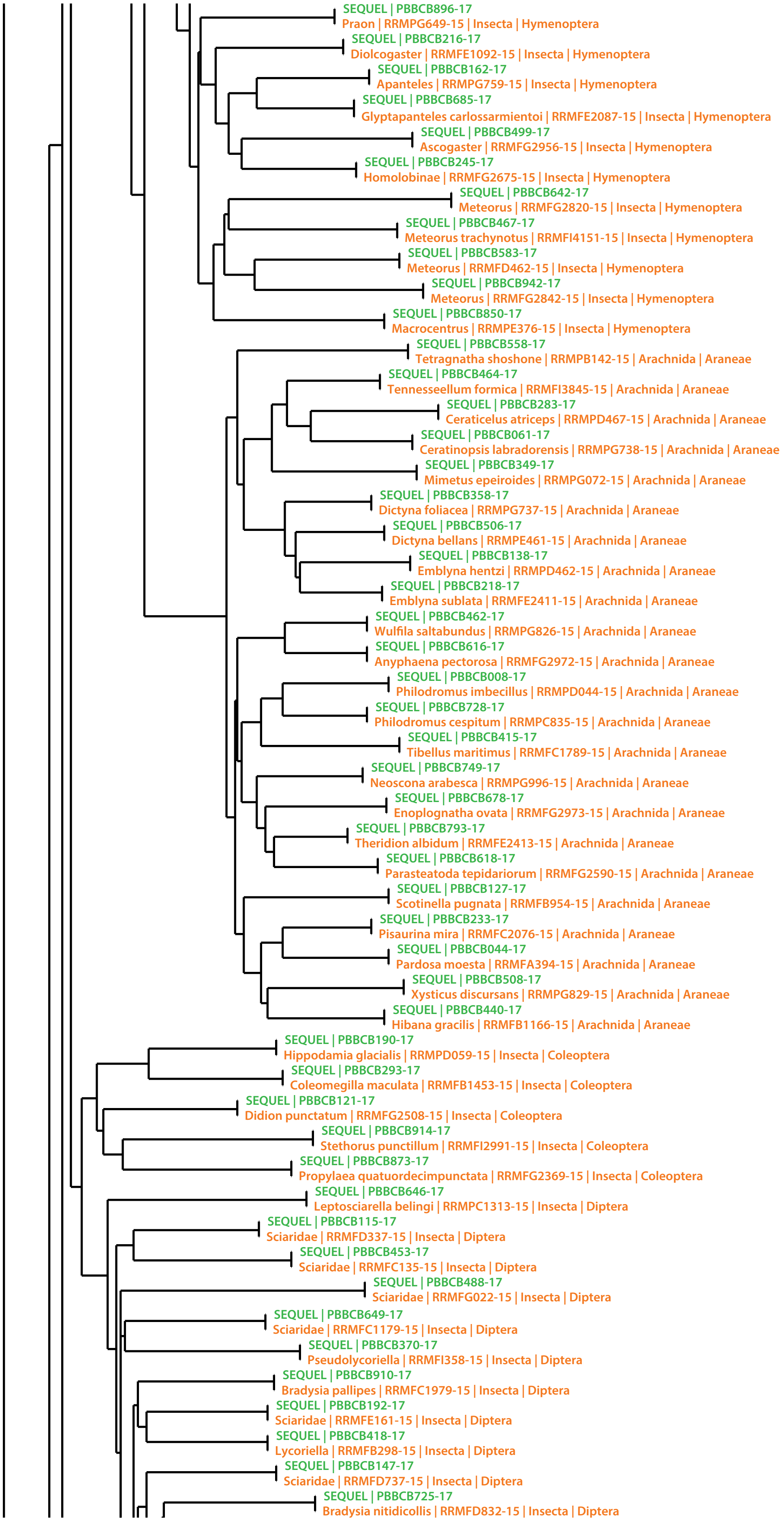

SEQUEL | PBBCB896-17
Praon | RRMPG649-15 | Insecta | Hymenoptera
SEQUEL | PBBCB216-17
Diolcogaster | RRMFE1092-15 | Insecta | Hymenoptera
SEQUEL | PBBCB162-17
Apanteles | RRMPG759-15 | Insecta | Hymenoptera
SEQUEL | PBBCB685-17
Glyptapanteles carlossarmientoi | RRMFE2087-15 | Insecta | Hymenoptera
SEQUEL | PBBCB499-17
Ascogaster | RRMFG2956-15 | Insecta | Hymenoptera
SEQUEL | PBBCB245-17
Homolobinae | RRMFG2675-15 | Insecta | Hymenoptera
SEQUEL | PBBCB642-17
Meteorus | RRMFG2820-15 | Insecta | Hymenoptera
SEQUEL | PBBCB467-17
Meteorus trachynotus | RRMFI4151-15 | Insecta | Hymenoptera
SEQUEL | PBBCB583-17
Meteorus | RRMFD462-15 | Insecta | Hymenoptera
SEQUEL | PBBCB942-17
Meteorus | RRMFG2842-15 | Insecta | Hymenoptera
SEQUEL | PBBCB850-17
Macrocentrus | RRMPE376-15 | Insecta | Hymenoptera
SEQUEL | PBBCB558-17
Tetragnatha shoshone | RRMPB142-15 | Arachnida | Araneae
SEQUEL | PBBCB464-17
Tennesseellum formica | RRMFI3845-15 | Arachnida | Araneae
SEQUEL | PBBCB283-17
Ceraticelus atriceps | RRMPD467-15 | Arachnida | Araneae
SEQUEL | PBBCB061-17
Ceratinopsis labradorensis | RRMPG738-15 | Arachnida | Araneae
SEQUEL | PBBCB349-17
Mimetus epeiroides | RRMPG072-15 | Arachnida | Araneae
SEQUEL | PBBCB358-17
Dictyna foliacea | RRMPG737-15 | Arachnida | Araneae
SEQUEL | PBBCB506-17
Dictyna bellans | RRMPE461-15 | Arachnida | Araneae
SEQUEL | PBBCB138-17
Emblyna hentzi | RRMPD462-15 | Arachnida | Araneae
SEQUEL | PBBCB218-17
Emblyna sublata | RRMFE2411-15 | Arachnida | Araneae
SEQUEL | PBBCB462-17
Wulfila saltabundus | RRMPG826-15 | Arachnida | Araneae
SEQUEL | PBBCB616-17
Anyphaena pectorosa | RRMFG2972-15 | Arachnida | Araneae
SEQUEL | PBBCB008-17
Philodromus imbecillus | RRMPD044-15 | Arachnida | Araneae
SEQUEL | PBBCB728-17
Philodromus cespitum | RRMPC835-15 | Arachnida | Araneae
SEQUEL | PBBCB415-17
Tibellus maritimus | RRMFC1789-15 | Arachnida | Araneae
SEQUEL | PBBCB749-17
Neoscona arabesca | RRMPG996-15 | Arachnida | Araneae
SEQUEL | PBBCB678-17
Enoplognatha ovata | RRMFG2973-15 | Arachnida | Araneae
SEQUEL | PBBCB793-17
Theridion albidum | RRMFE2413-15 | Arachnida | Araneae
SEQUEL | PBBCB618-17
Parasteatoda tepidariorum | RRMFG2590-15 | Arachnida | Araneae
SEQUEL | PBBCB127-17
Scotinella pugnata | RRMFB954-15 | Arachnida | Araneae
SEQUEL | PBBCB233-17
Pisaurina mira | RRMFC2076-15 | Arachnida | Araneae
SEQUEL | PBBCB044-17
Pardosa moesta | RRMFA394-15 | Arachnida | Araneae
SEQUEL | PBBCB508-17
Xysticus discursans | RRMPG829-15 | Arachnida | Araneae
SEQUEL | PBBCB440-17
Hibana gracilis | RRMFB1166-15 | Arachnida | Araneae
SEQUEL | PBBCB190-17
Hippodamia glacialis | RRMPD059-15 | Insecta | Coleoptera
SEQUEL | PBBCB293-17
Coleomegilla maculata | RRMFB1453-15 | Insecta | Coleoptera
SEQUEL | PBBCB121-17
Didion punctatum | RRMFG2508-15 | Insecta | Coleoptera
SEQUEL | PBBCB914-17
Stethorus punctillum | RRMFI2991-15 | Insecta | Coleoptera
SEQUEL | PBBCB873-17
Propylaea quatuordecimpunctata | RRMFG2369-15 | Insecta | Coleoptera
SEQUEL | PBBCB646-17
Leptosciarella belingi | RRMPC1313-15 | Insecta | Diptera
SEQUEL | PBBCB115-17
Sciaridae | RRMFD337-15 | Insecta | Diptera
SEQUEL | PBBCB453-17
Sciaridae | RRMFC135-15 | Insecta | Diptera
SEQUEL | PBBCB488-17
Sciaridae | RRMFG022-15 | Insecta | Diptera
SEQUEL | PBBCB649-17
Sciaridae | RRMFC1179-15 | Insecta | Diptera
SEQUEL | PBBCB370-17
Pseudolycoriella | RRMFI358-15 | Insecta | Diptera
SEQUEL | PBBCB910-17
Bradysia pallipes | RRMFC1979-15 | Insecta | Diptera
SEQUEL | PBBCB192-17
Sciaridae | RRMFE161-15 | Insecta | Diptera
SEQUEL | PBBCB418-17
Lycoriella | RRMFB298-15 | Insecta | Diptera
SEQUEL | PBBCB147-17
Sciaridae | RRMFD737-15 | Insecta | Diptera
SEQUEL | PBBCB725-17
Bradysia nitidicollis | RRMFD832-15 | Insecta | Diptera

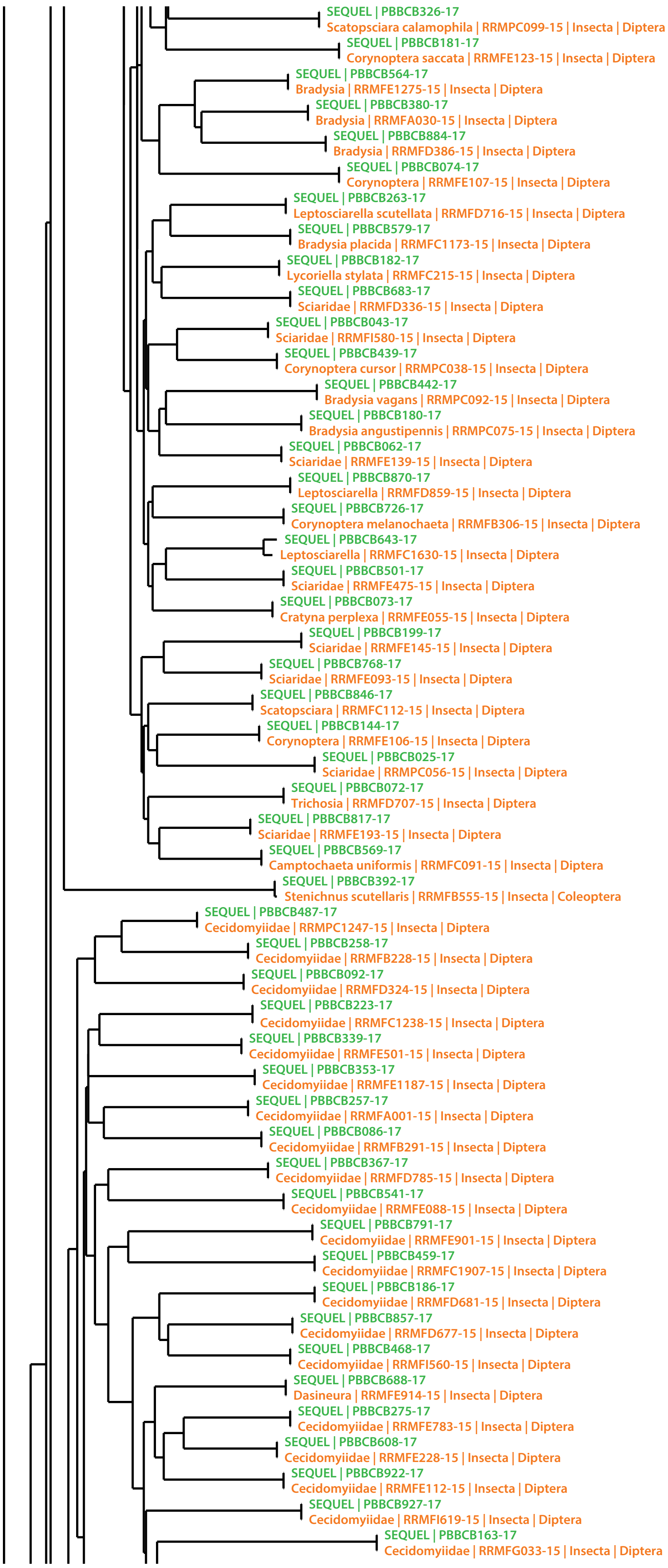

SEQUEL | PBBCB326-17
Scatopsciara calamophila | RRMPC099-15 | Insecta | Diptera
SEQUEL | PBBCB181-17
Corynoptera saccata | RRMFE123-15 | Insecta | Diptera
SEQUEL | PBBCB564-17
Bradysia | RRMFE1275-15 | Insecta | Diptera
SEQUEL | PBBCB380-17
Bradysia | RRMFA030-15 | Insecta | Diptera
SEQUEL | PBBCB884-17
Bradysia | RRMFD386-15 | Insecta | Diptera
SEQUEL | PBBCB074-17
Corynoptera | RRMFE107-15 | Insecta | Diptera
SEQUEL | PBBCB263-17
Leptosciarella scutellata | RRMFD716-15 | Insecta | Diptera
SEQUEL | PBBCB579-17
Bradysia placida | RRMFC1173-15 | Insecta | Diptera
SEQUEL | PBBCB182-17
Lycoriella stylata | RRMFC215-15 | Insecta | Diptera
SEQUEL | PBBCB683-17
Sciaridae | RRMFD336-15 | Insecta | Diptera
SEQUEL | PBBCB043-17
Sciaridae | RRMFI580-15 | Insecta | Diptera
SEQUEL | PBBCB439-17
Corynoptera cursor | RRMPC038-15 | Insecta | Diptera
SEQUEL | PBBCB442-17
Bradysia vagans | RRMPC092-15 | Insecta | Diptera
SEQUEL | PBBCB180-17
Bradysia angustipennis | RRMPC075-15 | Insecta | Diptera
SEQUEL | PBBCB062-17
Sciaridae | RRMFE139-15 | Insecta | Diptera
SEQUEL | PBBCB870-17
Leptosciarella | RRMFD859-15 | Insecta | Diptera
SEQUEL | PBBCB726-17
Corynoptera melanochaeta | RRMFB306-15 | Insecta | Diptera
SEQUEL | PBBCB643-17
Leptosciarella | RRMFC1630-15 | Insecta | Diptera
SEQUEL | PBBCB501-17
Sciaridae | RRMFE475-15 | Insecta | Diptera
SEQUEL | PBBCB073-17
Cratyna perplexa | RRMFE055-15 | Insecta | Diptera
SEQUEL | PBBCB199-17
Sciaridae | RRMFE145-15 | Insecta | Diptera
SEQUEL | PBBCB768-17
Sciaridae | RRMFE093-15 | Insecta | Diptera
SEQUEL | PBBCB846-17
Scatopsciara | RRMFC112-15 | Insecta | Diptera
SEQUEL | PBBCB144-17
Corynoptera | RRMFE106-15 | Insecta | Diptera
SEQUEL | PBBCB025-17
Sciaridae | RRMPC056-15 | Insecta | Diptera
SEQUEL | PBBCB072-17
Trichosia | RRMFD707-15 | Insecta | Diptera
SEQUEL | PBBCB817-17
Sciaridae | RRMFE193-15 | Insecta | Diptera
SEQUEL | PBBCB569-17
Camptochaeta uniformis | RRMFC091-15 | Insecta | Diptera
SEQUEL | PBBCB392-17
Stenichnus scutellaris | RRMFB555-15 | Insecta | Coleoptera
SEQUEL | PBBCB487-17
Cecidomyiidae | RRMPC1247-15 | Insecta | Diptera
SEQUEL | PBBCB258-17
Cecidomyiidae | RRMFB228-15 | Insecta | Diptera
SEQUEL | PBBCB092-17
Cecidomyiidae | RRMFD324-15 | Insecta | Diptera
SEQUEL | PBBCB223-17
Cecidomyiidae | RRMFC1238-15 | Insecta | Diptera
SEQUEL | PBBCB339-17
Cecidomyiidae | RRMFE501-15 | Insecta | Diptera
SEQUEL | PBBCB353-17
Cecidomyiidae | RRMFE1187-15 | Insecta | Diptera
SEQUEL | PBBCB257-17
Cecidomyiidae | RRMFA001-15 | Insecta | Diptera
SEQUEL | PBBCB086-17
Cecidomyiidae | RRMFB291-15 | Insecta | Diptera
SEQUEL | PBBCB367-17
Cecidomyiidae | RRMFD785-15 | Insecta | Diptera
SEQUEL | PBBCB541-17
Cecidomyiidae | RRMFE088-15 | Insecta | Diptera
SEQUEL | PBBCB791-17
Cecidomyiidae | RRMFE901-15 | Insecta | Diptera
SEQUEL | PBBCB459-17
Cecidomyiidae | RRMFC1907-15 | Insecta | Diptera
SEQUEL | PBBCB186-17
Cecidomyiidae | RRMFD681-15 | Insecta | Diptera
SEQUEL | PBBCB857-17
Cecidomyiidae | RRMFD677-15 | Insecta | Diptera
SEQUEL | PBBCB468-17
Cecidomyiidae | RRMFI560-15 | Insecta | Diptera
SEQUEL | PBBCB688-17
Dasineura | RRMFE914-15 | Insecta | Diptera
SEQUEL | PBBCB275-17
Cecidomyiidae | RRMFE783-15 | Insecta | Diptera
SEQUEL | PBBCB608-17
Cecidomyiidae | RRMFE228-15 | Insecta | Diptera
SEQUEL | PBBCB922-17
Cecidomyiidae | RRMFE112-15 | Insecta | Diptera
SEQUEL | PBBCB927-17
Cecidomyiidae | RRMFI619-15 | Insecta | Diptera
SEQUEL | PBBCB163-17
Cecidomyiidae | RRMFG033-15 | Insecta | Diptera

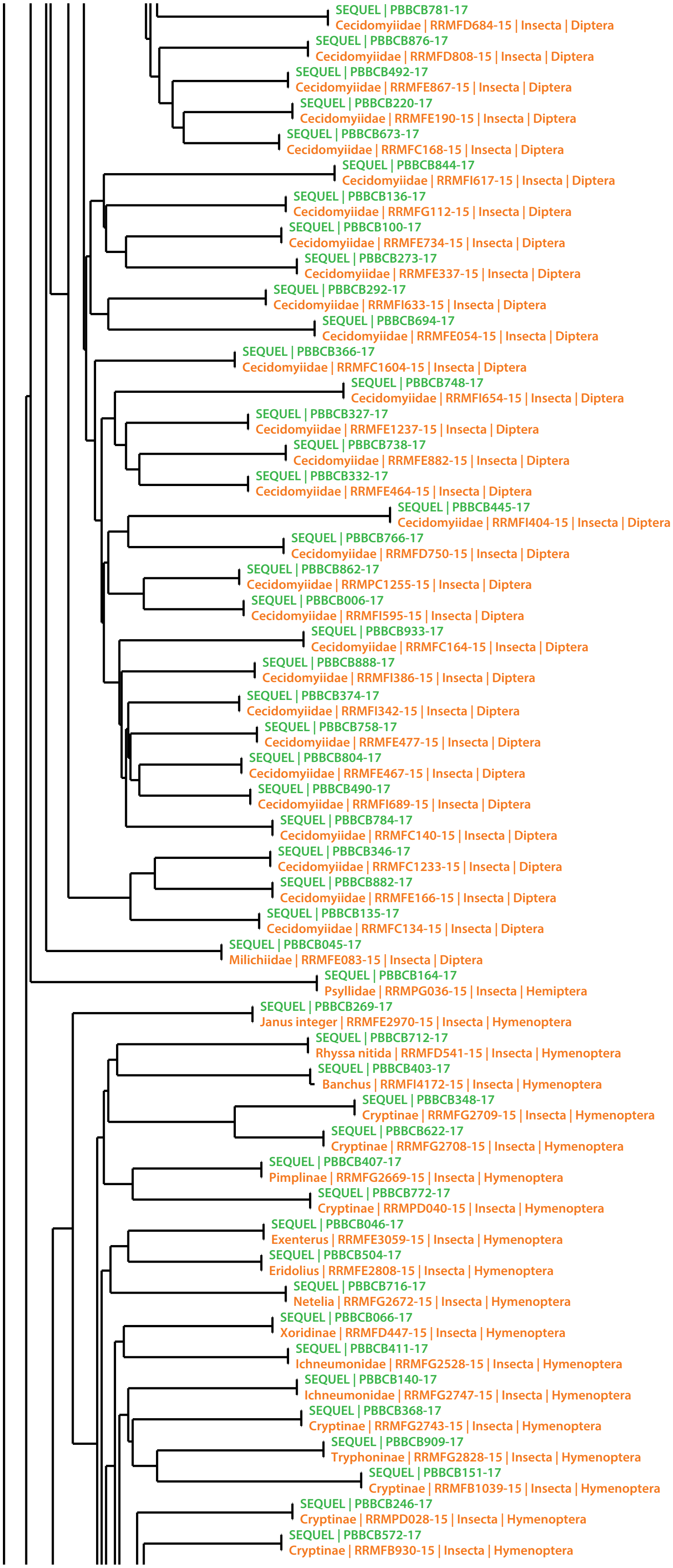
SEQUEL | PBBCB781-17
Cecidomyiidae | RRMFD684-15 | Insecta | Diptera
SEQUEL | PBBCB876-17
Cecidomyiidae | RRMFD808-15 | Insecta | Diptera
SEQUEL | PBBCB492-17
Cecidomyiidae | RRMFE867-15 | Insecta | Diptera
SEQUEL | PBBCB220-17
Cecidomyiidae | RRMFE190-15 | Insecta | Diptera
SEQUEL | PBBCB673-17
Cecidomyiidae | RRMFC168-15 | Insecta | Diptera
SEQUEL | PBBCB844-17
Cecidomyiidae | RRMFI617-15 | Insecta | Diptera
SEQUEL | PBBCB136-17
Cecidomyiidae | RRMFG112-15 | Insecta | Diptera
SEQUEL | PBBCB100-17
Cecidomyiidae | RRMFE734-15 | Insecta | Diptera
SEQUEL | PBBCB273-17
Cecidomyiidae | RRMFE337-15 | Insecta | Diptera
SEQUEL | PBBCB292-17
Cecidomyiidae | RRMFI633-15 | Insecta | Diptera
SEQUEL | PBBCB694-17
Cecidomyiidae | RRMFE054-15 | Insecta | Diptera
SEQUEL | PBBCB366-17
Cecidomyiidae | RRMFC1604-15 | Insecta | Diptera
SEQUEL | PBBCB748-17
Cecidomyiidae | RRMFI654-15 | Insecta | Diptera
SEQUEL | PBBCB327-17
Cecidomyiidae | RRMFE1237-15 | Insecta | Diptera
SEQUEL | PBBCB738-17
Cecidomyiidae | RRMFE882-15 | Insecta | Diptera
SEQUEL | PBBCB332-17
Cecidomyiidae | RRMFE464-15 | Insecta | Diptera
SEQUEL | PBBCB445-17
Cecidomyiidae | RRMFI404-15 | Insecta | Diptera
SEQUEL | PBBCB766-17
Cecidomyiidae | RRMFD750-15 | Insecta | Diptera
SEQUEL | PBBCB862-17
Cecidomyiidae | RRMPC1255-15 | Insecta | Diptera
SEQUEL | PBBCB006-17
Cecidomyiidae | RRMFI595-15 | Insecta | Diptera
SEQUEL | PBBCB933-17
Cecidomyiidae | RRMFC164-15 | Insecta | Diptera
SEQUEL | PBBCB888-17
Cecidomyiidae | RRMFI386-15 | Insecta | Diptera
SEQUEL | PBBCB374-17
Cecidomyiidae | RRMFI342-15 | Insecta | Diptera
SEQUEL | PBBCB758-17
Cecidomyiidae | RRMFE477-15 | Insecta | Diptera
SEQUEL | PBBCB804-17
Cecidomyiidae | RRMFE467-15 | Insecta | Diptera
SEQUEL | PBBCB490-17
Cecidomyiidae | RRMFI689-15 | Insecta | Diptera
SEQUEL | PBBCB784-17
Cecidomyiidae | RRMFC140-15 | Insecta | Diptera
SEQUEL | PBBCB346-17
Cecidomyiidae | RRMFC1233-15 | Insecta | Diptera
SEQUEL | PBBCB882-17
Cecidomyiidae | RRMFE166-15 | Insecta | Diptera
SEQUEL | PBBCB135-17
Cecidomyiidae | RRMFC134-15 | Insecta | Diptera
SEQUEL | PBBCB045-17
Milichiidae | RRMFE083-15 | Insecta | Diptera
SEQUEL | PBBCB164-17
Psyllidae | RRMPG036-15 | Insecta | Hemiptera
SEQUEL | PBBCB269-17
Janus integer | RRMFE2970-15 | Insecta | Hymenoptera
SEQUEL | PBBCB712-17
Rhyssa nitida | RRMFD541-15 | Insecta | Hymenoptera
SEQUEL | PBBCB403-17
Banchus | RRMFI4172-15 | Insecta | Hymenoptera
SEQUEL | PBBCB348-17
Cryptinae | RRMFG2709-15 | Insecta | Hymenoptera
SEQUEL | PBBCB622-17
Cryptinae | RRMFG2708-15 | Insecta | Hymenoptera
SEQUEL | PBBCB407-17
Pimplinae | RRMFG2669-15 | Insecta | Hymenoptera
SEQUEL | PBBCB772-17
Cryptinae | RRMPD040-15 | Insecta | Hymenoptera
SEQUEL | PBBCB046-17
Exenterus | RRMFE3059-15 | Insecta | Hymenoptera
SEQUEL | PBBCB504-17
Eridolius | RRMFE2808-15 | Insecta | Hymenoptera
SEQUEL | PBBCB716-17
Netelia | RRMFG2672-15 | Insecta | Hymenoptera
SEQUEL | PBBCB066-17
Xoridinae | RRMFD447-15 | Insecta | Hymenoptera
SEQUEL | PBBCB411-17
Ichneumonidae | RRMFG2528-15 | Insecta | Hymenoptera
SEQUEL | PBBCB140-17
Ichneumonidae | RRMFG2747-15 | Insecta | Hymenoptera
SEQUEL | PBBCB368-17
Cryptinae | RRMFG2743-15 | Insecta | Hymenoptera
SEQUEL | PBBCB909-17
Tryphoninae | RRMFG2828-15 | Insecta | Hymenoptera
SEQUEL | PBBCB151-17
Cryptinae | RRMFB1039-15 | Insecta | Hymenoptera
SEQUEL | PBBCB246-17
Cryptinae | RRMPD028-15 | Insecta | Hymenoptera
SEQUEL | PBBCB572-17
Cryptinae | RRMFB930-15 | Insecta | Hymenoptera

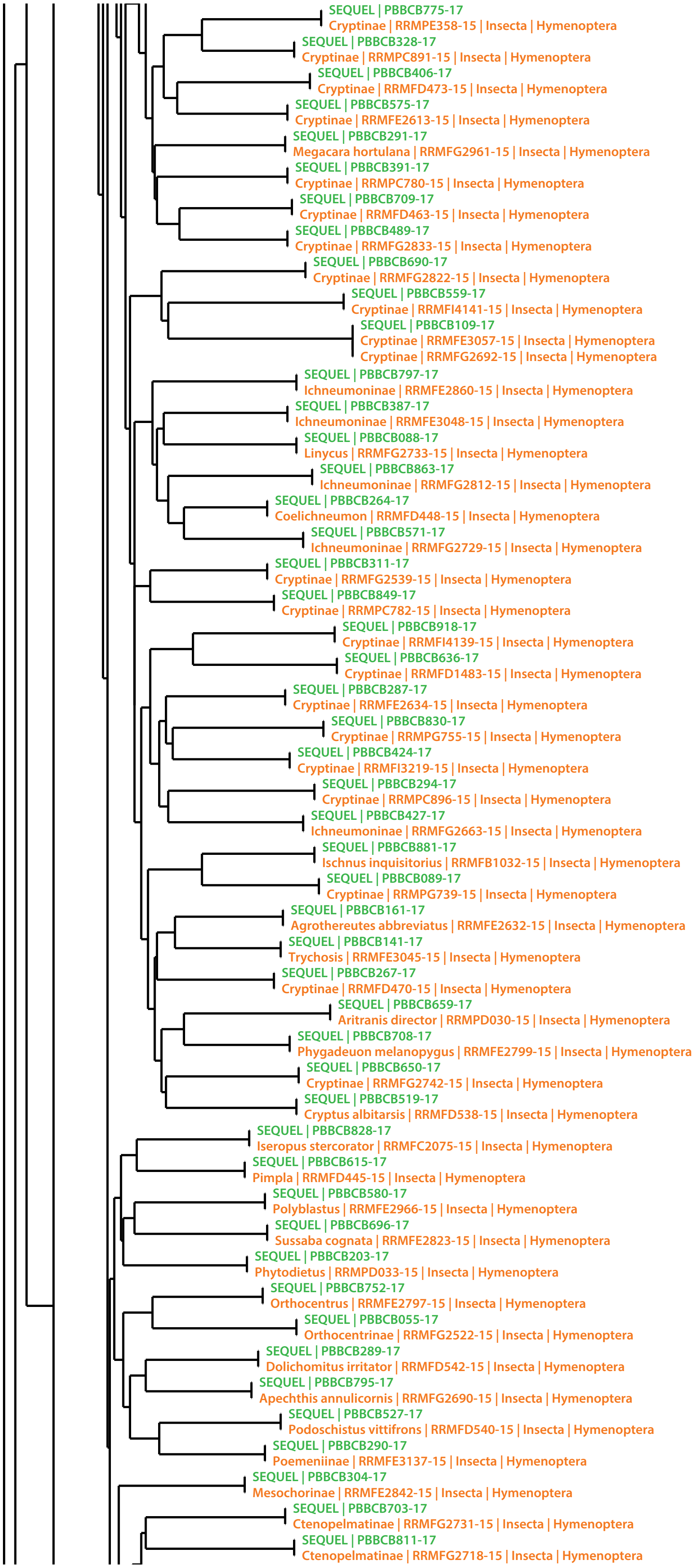

SEQUEL | PBBCB775-17
Cryptinae | RRMPE358-15 | Insecta | Hymenoptera
SEQUEL | PBBCB328-17
Cryptinae | RRMPC891-15 | Insecta | Hymenoptera
SEQUEL | PBBCB406-17
Cryptinae | RRMFD473-15 | Insecta | Hymenoptera
SEQUEL | PBBCB575-17
Cryptinae | RRMFE2613-15 | Insecta | Hymenoptera
SEQUEL | PBBCB291-17
Megacara hortulana | RRMFG2961-15 | Insecta | Hymenoptera
SEQUEL | PBBCB391-17
Cryptinae | RRMPC780-15 | Insecta | Hymenoptera
SEQUEL | PBBCB709-17
Cryptinae | RRMFD463-15 | Insecta | Hymenoptera
SEQUEL | PBBCB489-17
Cryptinae | RRMFG2833-15 | Insecta | Hymenoptera
SEQUEL | PBBCB690-17
Cryptinae | RRMFG2822-15 | Insecta | Hymenoptera
SEQUEL | PBBCB559-17
Cryptinae | RRMFI4141-15 | Insecta | Hymenoptera
SEQUEL | PBBCB109-17
Cryptinae | RRMFE3057-15 | Insecta | Hymenoptera
Cryptinae | RRMFG2692-15 | Insecta | Hymenoptera
SEQUEL | PBBCB797-17
Ichneumoninae | RRMFE2860-15 | Insecta | Hymenoptera
SEQUEL | PBBCB387-17
Ichneumoninae | RRMFE3048-15 | Insecta | Hymenoptera
SEQUEL | PBBCB088-17
Linycus | RRMFG2733-15 | Insecta | Hymenoptera
SEQUEL | PBBCB863-17
Ichneumoninae | RRMFG2812-15 | Insecta | Hymenoptera
SEQUEL | PBBCB264-17
Coelichneumon | RRMFD448-15 | Insecta | Hymenoptera
SEQUEL | PBBCB571-17
Ichneumoninae | RRMFG2729-15 | Insecta | Hymenoptera
SEQUEL | PBBCB311-17
Cryptinae | RRMFG2539-15 | Insecta | Hymenoptera
SEQUEL | PBBCB849-17
Cryptinae | RRMPC782-15 | Insecta | Hymenoptera
SEQUEL | PBBCB918-17
Cryptinae | RRMFI4139-15 | Insecta | Hymenoptera
SEQUEL | PBBCB636-17
Cryptinae | RRMFD1483-15 | Insecta | Hymenoptera
SEQUEL | PBBCB287-17
Cryptinae | RRMFE2634-15 | Insecta | Hymenoptera
SEQUEL | PBBCB830-17
Cryptinae | RRMPG755-15 | Insecta | Hymenoptera
SEQUEL | PBBCB424-17
Cryptinae | RRMFI3219-15 | Insecta | Hymenoptera
SEQUEL | PBBCB294-17
Cryptinae | RRMPC896-15 | Insecta | Hymenoptera
SEQUEL | PBBCB427-17
Ichneumoninae | RRMFG2663-15 | Insecta | Hymenoptera
SEQUEL | PBBCB881-17
Ischnus inquisitorius | RRMFB1032-15 | Insecta | Hymenoptera
SEQUEL | PBBCB089-17
Cryptinae | RRMPG739-15 | Insecta | Hymenoptera
SEQUEL | PBBCB161-17
Agrothereutes abbreviatus | RRMFE2632-15 | Insecta | Hymenoptera
SEQUEL | PBBCB141-17
Trychosis | RRMFE3045-15 | Insecta | Hymenoptera
SEQUEL | PBBCB267-17
Cryptinae | RRMFD470-15 | Insecta | Hymenoptera
SEQUEL | PBBCB659-17
Aritranis director | RRMPD030-15 | Insecta | Hymenoptera
SEQUEL | PBBCB708-17
Phygadeuon melanopygus | RRMFE2799-15 | Insecta | Hymenoptera
SEQUEL | PBBCB650-17
Cryptinae | RRMFG2742-15 | Insecta | Hymenoptera
SEQUEL | PBBCB519-17
Cryptus albitarsis | RRMFD538-15 | Insecta | Hymenoptera
SEQUEL | PBBCB828-17
Iseropus stercorator | RRMFC2075-15 | Insecta | Hymenoptera
SEQUEL | PBBCB615-17
Pimpla | RRMFD445-15 | Insecta | Hymenoptera
SEQUEL | PBBCB580-17
Polyblastus | RRMFE2966-15 | Insecta | Hymenoptera
SEQUEL | PBBCB696-17
Sussaba cognata | RRMFE2823-15 | Insecta | Hymenoptera
SEQUEL | PBBCB203-17
Phytodietus | RRMPD033-15 | Insecta | Hymenoptera
SEQUEL | PBBCB752-17
Orthocentrus | RRMFE2797-15 | Insecta | Hymenoptera
SEQUEL | PBBCB055-17
Orthocentrinae | RRMFG2522-15 | Insecta | Hymenoptera
SEQUEL | PBBCB289-17
Dolichomitus irritator | RRMFD542-15 | Insecta | Hymenoptera
SEQUEL | PBBCB795-17
Apechthis annulicornis | RRMFG2690-15 | Insecta | Hymenoptera
SEQUEL | PBBCB527-17
Podoschistus vittifrons | RRMFD540-15 | Insecta | Hymenoptera
SEQUEL | PBBCB290-17
Poemeniinae | RRMFE3137-15 | Insecta | Hymenoptera
SEQUEL | PBBCB304-17
Mesochorinae | RRMFE2842-15 | Insecta | Hymenoptera
SEQUEL | PBBCB703-17
Ctenopelmatinae | RRMFG2731-15 | Insecta | Hymenoptera
SEQUEL | PBBCB811-17
Ctenopelmatinae | RRMFG2718-15 | Insecta | Hymenoptera

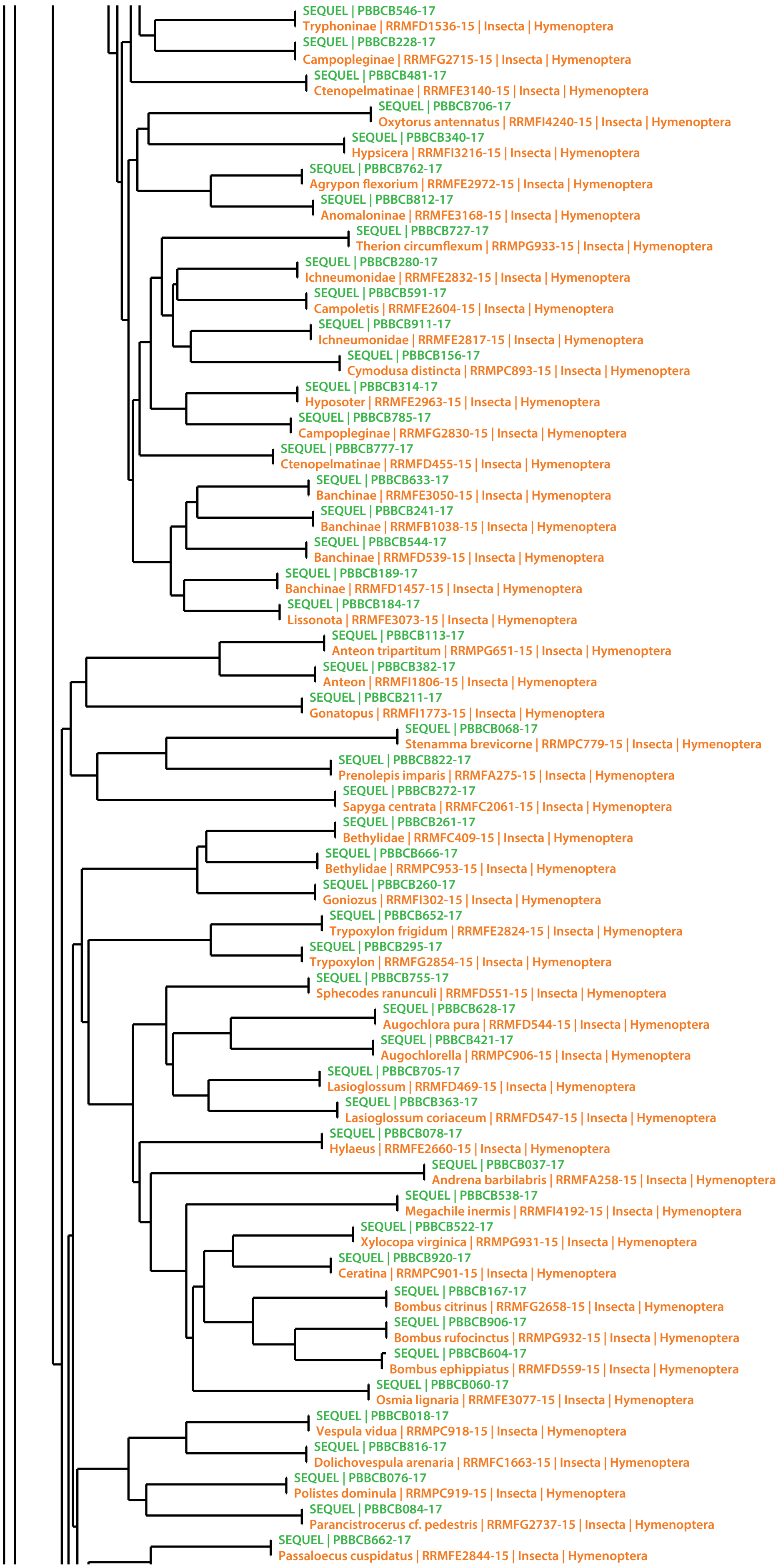
SEQUEL | PBBCB546-17
Tryphoninae | RRMFD1536-15 | Insecta | Hymenoptera
SEQUEL | PBBCB228-17
Campopleginae | RRMFG2715-15 | Insecta | Hymenoptera
SEQUEL | PBBCB481-17
Ctenopelmatinae | RRMFE3140-15 | Insecta | Hymenoptera
SEQUEL | PBBCB706-17
Oxytorus antennatus | RRMFI4240-15 | Insecta | Hymenoptera
SEQUEL | PBBCB340-17
Hypsicera | RRMFI3216-15 | Insecta | Hymenoptera
SEQUEL | PBBCB762-17
Agrypon flexorium | RRMFE2972-15 | Insecta | Hymenoptera
SEQUEL | PBBCB812-17
Anomaloninae | RRMFE3168-15 | Insecta | Hymenoptera
SEQUEL | PBBCB727-17
Therion circumflexum | RRMPG933-15 | Insecta | Hymenoptera
SEQUEL | PBBCB280-17
Ichneumonidae | RRMFE2832-15 | Insecta | Hymenoptera
SEQUEL | PBBCB591-17
Campoletis | RRMFE2604-15 | Insecta | Hymenoptera
SEQUEL | PBBCB911-17
Ichneumonidae | RRMFE2817-15 | Insecta | Hymenoptera
SEQUEL | PBBCB156-17
Cymodusa distincta | RRMPC893-15 | Insecta | Hymenoptera
SEQUEL | PBBCB314-17
Hyposoter | RRMFE2963-15 | Insecta | Hymenoptera
SEQUEL | PBBCB785-17
Campopleginae | RRMFG2830-15 | Insecta | Hymenoptera
SEQUEL | PBBCB777-17
Ctenopelmatinae | RRMFD455-15 | Insecta | Hymenoptera
SEQUEL | PBBCB633-17
Banchinae | RRMFE3050-15 | Insecta | Hymenoptera
SEQUEL | PBBCB241-17
Banchinae | RRMFB1038-15 | Insecta | Hymenoptera
SEQUEL | PBBCB544-17
Banchinae | RRMFD539-15 | Insecta | Hymenoptera
SEQUEL | PBBCB189-17
Banchinae | RRMFD1457-15 | Insecta | Hymenoptera
SEQUEL | PBBCB184-17
Lissonota | RRMFE3073-15 | Insecta | Hymenoptera
SEQUEL | PBBCB113-17
Anteon tripartitum | RRMPG651-15 | Insecta | Hymenoptera
SEQUEL | PBBCB382-17
Anteon | RRMFI1806-15 | Insecta | Hymenoptera
SEQUEL | PBBCB211-17
Gonatopus | RRMFI1773-15 | Insecta | Hymenoptera
SEQUEL | PBBCB068-17
Stenamma brevicorne | RRMPC779-15 | Insecta | Hymenoptera
SEQUEL | PBBCB822-17
Prenolepis imparis | RRMFA275-15 | Insecta | Hymenoptera
SEQUEL | PBBCB272-17
Sapyga centrata | RRMFC2061-15 | Insecta | Hymenoptera
SEQUEL | PBBCB261-17
Bethylidae | RRMFC409-15 | Insecta | Hymenoptera
SEQUEL | PBBCB666-17
Bethylidae | RRMPC953-15 | Insecta | Hymenoptera
SEQUEL | PBBCB260-17
Goniozus | RRMFI302-15 | Insecta | Hymenoptera
SEQUEL | PBBCB652-17
Trypoxylon frigidum | RRMFE2824-15 | Insecta | Hymenoptera
SEQUEL | PBBCB295-17
Trypoxylon | RRMFG2854-15 | Insecta | Hymenoptera
SEQUEL | PBBCB755-17
Sphecodes ranunculi | RRMFD551-15 | Insecta | Hymenoptera
SEQUEL | PBBCB628-17
Augochlora pura | RRMFD544-15 | Insecta | Hymenoptera
SEQUEL | PBBCB421-17
Augochlorella | RRMPC906-15 | Insecta | Hymenoptera
SEQUEL | PBBCB705-17
Lasioglossum | RRMFD469-15 | Insecta | Hymenoptera
SEQUEL | PBBCB363-17
Lasioglossum coriaceum | RRMFD547-15 | Insecta | Hymenoptera
SEQUEL | PBBCB078-17
Hylaeus | RRMFE2660-15 | Insecta | Hymenoptera
SEQUEL | PBBCB037-17
Andrena barbilabris | RRMFA258-15 | Insecta | Hymenoptera
SEQUEL | PBBCB538-17
Megachile inermis | RRMFI4192-15 | Insecta | Hymenoptera
SEQUEL | PBBCB522-17
Xylocopa virginica | RRMPG931-15 | Insecta | Hymenoptera
SEQUEL | PBBCB920-17
Ceratina | RRMPC901-15 | Insecta | Hymenoptera
SEQUEL | PBBCB167-17
Bombus citrinus | RRMFG2658-15 | Insecta | Hymenoptera
SEQUEL | PBBCB906-17
Bombus rufocinctus | RRMPG932-15 | Insecta | Hymenoptera
SEQUEL | PBBCB604-17
Bombus ephippiatus | RRMFD559-15 | Insecta | Hymenoptera
SEQUEL | PBBCB060-17
Osmia lignaria | RRMFE3077-15 | Insecta | Hymenoptera
SEQUEL | PBBCB018-17
Vespula vidua | RRMPC918-15 | Insecta | Hymenoptera
SEQUEL | PBBCB816-17
Dolichovespula arenaria | RRMFC1663-15 | Insecta | Hymenoptera
SEQUEL | PBBCB076-17
Polistes dominula | RRMPC919-15 | Insecta | Hymenoptera
SEQUEL | PBBCB084-17
Parancistrocerus cf. pedestris | RRMFG2737-15 | Insecta | Hymenoptera
SEQUEL | PBBCB662-17
Passaloecus cuspidatus | RRMFE2844-15 | Insecta | Hymenoptera

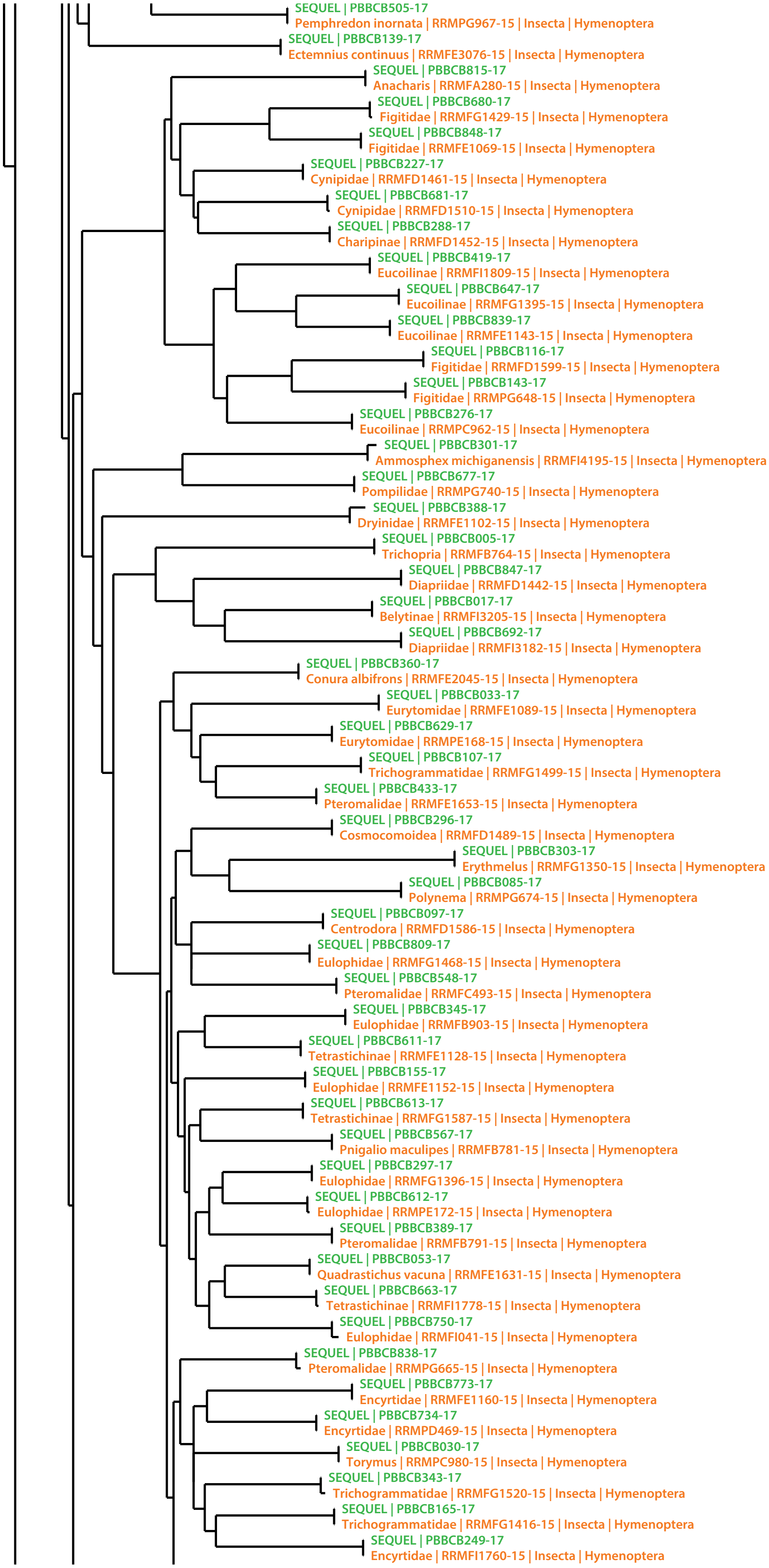

SEQUEL | PBBCB505-17
Pemphredon inornata | RRMPG967-15 | Insecta | Hymenoptera
SEQUEL | PBBCB139-17
Ectemnius continuus | RRMFE3076-15 | Insecta | Hymenoptera
SEQUEL | PBBCB815-17
Anacharis | RRMFA280-15 | Insecta | Hymenoptera
SEQUEL | PBBCB680-17
Figitidae | RRMFG1429-15 | Insecta | Hymenoptera
SEQUEL | PBBCB848-17
Figitidae | RRMFE1069-15 | Insecta | Hymenoptera
SEQUEL | PBBCB227-17
Cynipidae | RRMFD1461-15 | Insecta | Hymenoptera
SEQUEL | PBBCB681-17
Cynipidae | RRMFD1510-15 | Insecta | Hymenoptera
SEQUEL | PBBCB288-17
Charipinae | RRMFD1452-15 | Insecta | Hymenoptera
SEQUEL | PBBCB419-17
Eucoilinae | RRMFI1809-15 | Insecta | Hymenoptera
SEQUEL | PBBCB647-17
Eucoilinae | RRMFG1395-15 | Insecta | Hymenoptera
SEQUEL | PBBCB839-17
Eucoilinae | RRMFE1143-15 | Insecta | Hymenoptera
SEQUEL | PBBCB116-17
Figitidae | RRMFD1599-15 | Insecta | Hymenoptera
SEQUEL | PBBCB143-17
Figitidae | RRMPG648-15 | Insecta | Hymenoptera
SEQUEL | PBBCB276-17
Eucoilinae | RRMPC962-15 | Insecta | Hymenoptera
SEQUEL | PBBCB301-17
Ammosphex michiganensis | RRMFI4195-15 | Insecta | Hymenoptera
SEQUEL | PBBCB677-17
Pompilidae | RRMPG740-15 | Insecta | Hymenoptera
SEQUEL | PBBCB388-17
Dryinidae | RRMFE1102-15 | Insecta | Hymenoptera
SEQUEL | PBBCB005-17
Trichopria | RRMFB764-15 | Insecta | Hymenoptera
SEQUEL | PBBCB847-17
Diapriidae | RRMFD1442-15 | Insecta | Hymenoptera
SEQUEL | PBBCB017-17
Belytinae | RRMFI3205-15 | Insecta | Hymenoptera
SEQUEL | PBBCB692-17
Diapriidae | RRMFI3182-15 | Insecta | Hymenoptera
SEQUEL | PBBCB360-17
Conura albifrons | RRMFE2045-15 | Insecta | Hymenoptera
SEQUEL | PBBCB033-17
Eurytomidae | RRMFE1089-15 | Insecta | Hymenoptera
SEQUEL | PBBCB629-17
Eurytomidae | RRMPE168-15 | Insecta | Hymenoptera
SEQUEL | PBBCB107-17
Trichogrammatidae | RRMFG1499-15 | Insecta | Hymenoptera
SEQUEL | PBBCB433-17
Pteromalidae | RRMFE1653-15 | Insecta | Hymenoptera
SEQUEL | PBBCB296-17
Cosmocomoidea | RRMFD1489-15 | Insecta | Hymenoptera
SEQUEL | PBBCB303-17
Erythmelus | RRMFG1350-15 | Insecta | Hymenoptera
SEQUEL | PBBCB085-17
Polynema | RRMPG674-15 | Insecta | Hymenoptera
SEQUEL | PBBCB097-17
Centrodora | RRMFD1586-15 | Insecta | Hymenoptera
SEQUEL | PBBCB809-17
Eulophidae | RRMFG1468-15 | Insecta | Hymenoptera
SEQUEL | PBBCB548-17
Pteromalidae | RRMFC493-15 | Insecta | Hymenoptera
SEQUEL | PBBCB345-17
Eulophidae | RRMFB903-15 | Insecta | Hymenoptera
SEQUEL | PBBCB611-17
Tetrastichinae | RRMFE1128-15 | Insecta | Hymenoptera
SEQUEL | PBBCB155-17
Eulophidae | RRMFE1152-15 | Insecta | Hymenoptera
SEQUEL | PBBCB613-17
Tetrastichinae | RRMFG1587-15 | Insecta | Hymenoptera
SEQUEL | PBBCB567-17
Pnigalio maculipes | RRMFB781-15 | Insecta | Hymenoptera
SEQUEL | PBBCB297-17
Eulophidae | RRMFG1396-15 | Insecta | Hymenoptera
SEQUEL | PBBCB612-17
Eulophidae | RRMPE172-15 | Insecta | Hymenoptera
SEQUEL | PBBCB389-17
Pteromalidae | RRMFB791-15 | Insecta | Hymenoptera
SEQUEL | PBBCB053-17
Quadrastichus vacuna | RRMFE1631-15 | Insecta | Hymenoptera
SEQUEL | PBBCB663-17
Tetrastichinae | RRMFI1778-15 | Insecta | Hymenoptera
SEQUEL | PBBCB750-17
Eulophidae | RRMFI041-15 | Insecta | Hymenoptera
SEQUEL | PBBCB838-17
Pteromalidae | RRMPG665-15 | Insecta | Hymenoptera
SEQUEL | PBBCB773-17
Encyrtidae | RRMFE1160-15 | Insecta | Hymenoptera
SEQUEL | PBBCB734-17
Encyrtidae | RRMPD469-15 | Insecta | Hymenoptera
SEQUEL | PBBCB030-17
Torymus | RRMPC980-15 | Insecta | Hymenoptera
SEQUEL | PBBCB343-17
Trichogrammatidae | RRMFG1520-15 | Insecta | Hymenoptera
SEQUEL | PBBCB165-17
Trichogrammatidae | RRMFG1416-15 | Insecta | Hymenoptera
SEQUEL | PBBCB249-17
Encyrtidae | RRMFI1760-15 | Insecta | Hymenoptera

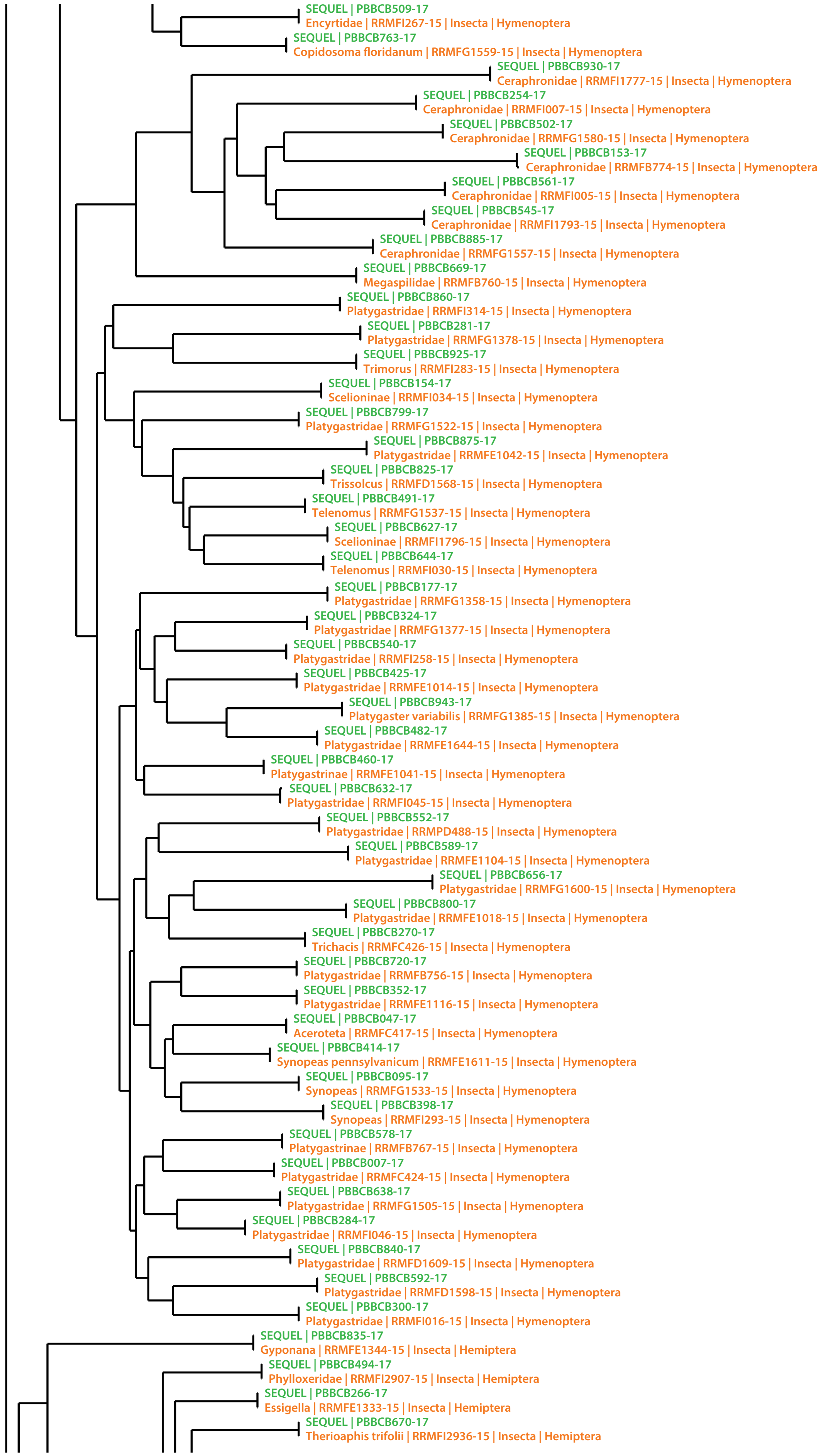
SEQUEL | PBBCB509-17
Encyrtidae | RRMFI267-15 | Insecta | Hymenoptera
SEQUEL | PBBCB763-17
Copidosoma floridanum | RRMFG1559-15 | Insecta | Hymenoptera
SEQUEL | PBBCB930-17
Ceraphronidae | RRMFI1777-15 | Insecta | Hymenoptera
SEQUEL | PBBCB254-17
Ceraphronidae | RRMFI007-15 | Insecta | Hymenoptera
SEQUEL | PBBCB502-17
Ceraphronidae | RRMFG1580-15 | Insecta | Hymenoptera
SEQUEL | PBBCB153-17
Ceraphronidae | RRMFB774-15 | Insecta | Hymenoptera
SEQUEL | PBBCB561-17
Ceraphronidae | RRMFI005-15 | Insecta | Hymenoptera
SEQUEL | PBBCB545-17
Ceraphronidae | RRMFI1793-15 | Insecta | Hymenoptera
SEQUEL | PBBCB885-17
Ceraphronidae | RRMFG1557-15 | Insecta | Hymenoptera
SEQUEL | PBBCB669-17
Megaspilidae | RRMFB760-15 | Insecta | Hymenoptera
SEQUEL | PBBCB860-17
Platygastridae | RRMFI314-15 | Insecta | Hymenoptera
SEQUEL | PBBCB281-17
Platygastridae | RRMFG1378-15 | Insecta | Hymenoptera
SEQUEL | PBBCB925-17
Trimorus | RRMFI283-15 | Insecta | Hymenoptera
SEQUEL | PBBCB154-17
Scelioninae | RRMFI034-15 | Insecta | Hymenoptera
SEQUEL | PBBCB799-17
Platygastridae | RRMFG1522-15 | Insecta | Hymenoptera
SEQUEL | PBBCB875-17
Platygastridae | RRMFE1042-15 | Insecta | Hymenoptera
SEQUEL | PBBCB825-17
Trissolcus | RRMFD1568-15 | Insecta | Hymenoptera
SEQUEL | PBBCB491-17
Telenomus | RRMFG1537-15 | Insecta | Hymenoptera
SEQUEL | PBBCB627-17
Scelioninae | RRMFI1796-15 | Insecta | Hymenoptera
SEQUEL | PBBCB644-17
Telenomus | RRMFI030-15 | Insecta | Hymenoptera
SEQUEL | PBBCB177-17
Platygastridae | RRMFG1358-15 | Insecta | Hymenoptera
SEQUEL | PBBCB324-17
Platygastridae | RRMFG1377-15 | Insecta | Hymenoptera
SEQUEL | PBBCB540-17
Platygastridae | RRMFI258-15 | Insecta | Hymenoptera
SEQUEL | PBBCB425-17
Platygastridae | RRMFE1014-15 | Insecta | Hymenoptera
SEQUEL | PBBCB943-17
Platygaster variabilis | RRMFG1385-15 | Insecta | Hymenoptera
SEQUEL | PBBCB482-17
Platygastridae | RRMFE1644-15 | Insecta | Hymenoptera
SEQUEL | PBBCB460-17
Platygastrinae | RRMFE1041-15 | Insecta | Hymenoptera
SEQUEL | PBBCB632-17
Platygastridae | RRMFI045-15 | Insecta | Hymenoptera
SEQUEL | PBBCB552-17
Platygastridae | RRMPD488-15 | Insecta | Hymenoptera
SEQUEL | PBBCB589-17
Platygastridae | RRMFE1104-15 | Insecta | Hymenoptera
SEQUEL | PBBCB656-17
Platygastridae | RRMFG1600-15 | Insecta | Hymenoptera
SEQUEL | PBBCB800-17
Platygastridae | RRMFE1018-15 | Insecta | Hymenoptera
SEQUEL | PBBCB270-17
Trichacis | RRMFC426-15 | Insecta | Hymenoptera
SEQUEL | PBBCB720-17
Platygastridae | RRMFB756-15 | Insecta | Hymenoptera
SEQUEL | PBBCB352-17
Platygastridae | RRMFE1116-15 | Insecta | Hymenoptera
SEQUEL | PBBCB047-17
Aceroteta | RRMFC417-15 | Insecta | Hymenoptera
SEQUEL | PBBCB414-17
Synopeas pennsylvanicum | RRMFE1611-15 | Insecta | Hymenoptera
SEQUEL | PBBCB095-17
Synopeas | RRMFG1533-15 | Insecta | Hymenoptera
SEQUEL | PBBCB398-17
Synopeas | RRMFI293-15 | Insecta | Hymenoptera
SEQUEL | PBBCB578-17
Platygastrinae | RRMFB767-15 | Insecta | Hymenoptera
SEQUEL | PBBCB007-17
Platygastridae | RRMFC424-15 | Insecta | Hymenoptera
SEQUEL | PBBCB638-17
Platygastridae | RRMFG1505-15 | Insecta | Hymenoptera
SEQUEL | PBBCB284-17
Platygastridae | RRMFI046-15 | Insecta | Hymenoptera
SEQUEL | PBBCB840-17
Platygastridae | RRMFD1609-15 | Insecta | Hymenoptera
SEQUEL | PBBCB592-17
Platygastridae | RRMFD1598-15 | Insecta | Hymenoptera
SEQUEL | PBBCB300-17
Platygastridae | RRMFI016-15 | Insecta | Hymenoptera
SEQUEL | PBBCB835-17
Gyponana | RRMFE1344-15 | Insecta | Hemiptera
SEQUEL | PBBCB494-17
Phylloxeridae | RRMFI2907-15 | Insecta | Hemiptera
SEQUEL | PBBCB266-17
Essigella | RRMFE1333-15 | Insecta | Hemiptera
SEQUEL | PBBCB670-17
Therioaphis trifolii | RRMFI2936-15 | Insecta | Hemiptera

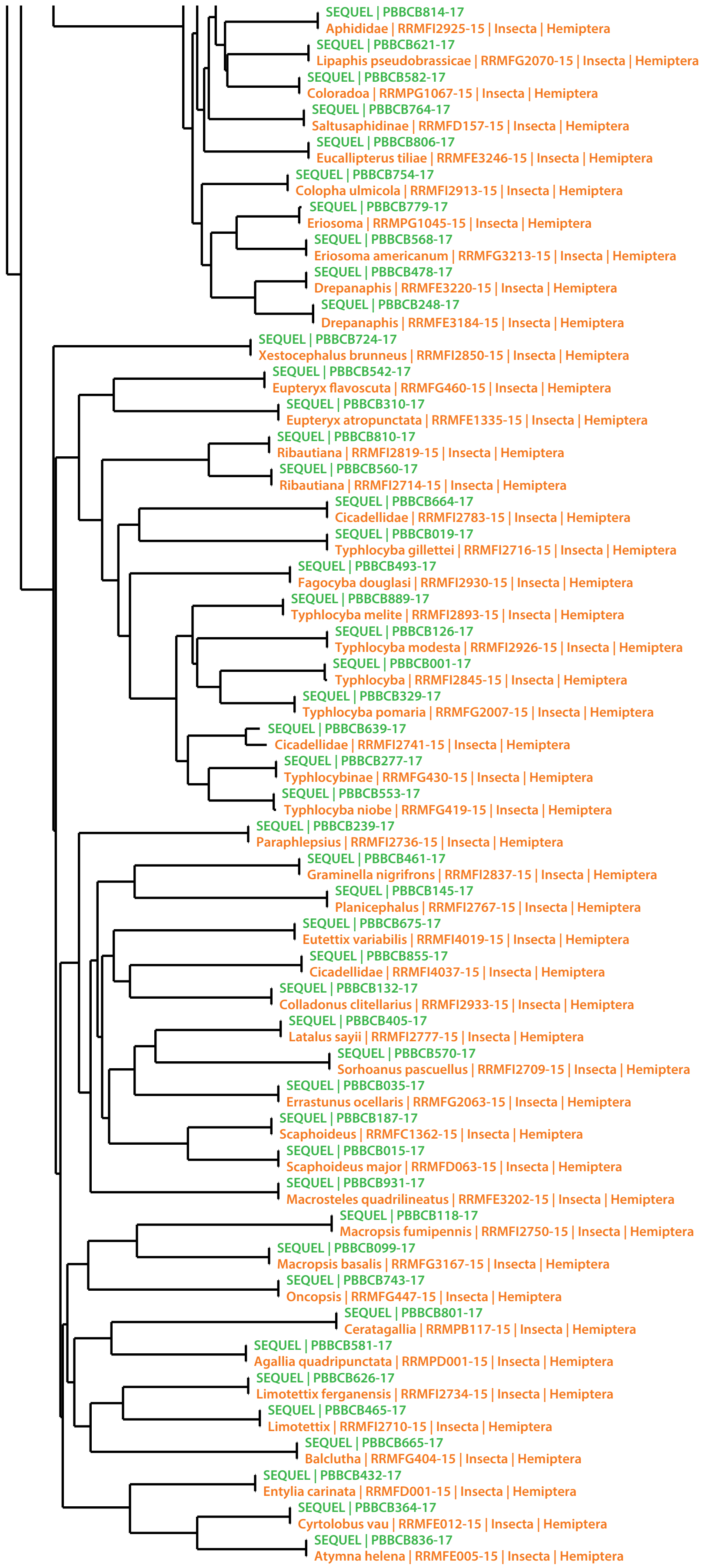
SEQUEL | PBBCB814-17
Aphididae | RRMFI2925-15 | Insecta | Hemiptera
SEQUEL | PBBCB621-17
Lipaphis pseudobrassicae | RRMFG2070-15 | Insecta | Hemiptera
SEQUEL | PBBCB582-17
Coloradoa | RRMPG1067-15 | Insecta | Hemiptera
SEQUEL | PBBCB764-17
Saltusaphidinae | RRMFD157-15 | Insecta | Hemiptera
SEQUEL | PBBCB806-17
Eucallipterus tiliae | RRMFE3246-15 | Insecta | Hemiptera
SEQUEL | PBBCB754-17
Colopha ulmicola | RRMFI2913-15 | Insecta | Hemiptera
SEQUEL | PBBCB779-17
Eriosoma | RRMPG1045-15 | Insecta | Hemiptera
SEQUEL | PBBCB568-17
Eriosoma americanum | RRMFG3213-15 | Insecta | Hemiptera
SEQUEL | PBBCB478-17
Drepanaphis | RRMFE3220-15 | Insecta | Hemiptera
SEQUEL | PBBCB248-17
Drepanaphis | RRMFE3184-15 | Insecta | Hemiptera
SEQUEL | PBBCB724-17
Xestocephalus brunneus | RRMFI2850-15 | Insecta | Hemiptera
SEQUEL | PBBCB542-17
Eupteryx flavoscuta | RRMFG460-15 | Insecta | Hemiptera
SEQUEL | PBBCB310-17
Eupteryx atropunctata | RRMFE1335-15 | Insecta | Hemiptera
SEQUEL | PBBCB810-17
Ribautiana | RRMFI2819-15 | Insecta | Hemiptera
SEQUEL | PBBCB560-17
Ribautiana | RRMFI2714-15 | Insecta | Hemiptera
SEQUEL | PBBCB664-17
Cicadellidae | RRMFI2783-15 | Insecta | Hemiptera
SEQUEL | PBBCB019-17
Typhlocyba gillettei | RRMFI2716-15 | Insecta | Hemiptera
SEQUEL | PBBCB493-17
Fagocyba douglasi | RRMFI2930-15 | Insecta | Hemiptera
SEQUEL | PBBCB889-17
Typhlocyba melite | RRMFI2893-15 | Insecta | Hemiptera
SEQUEL | PBBCB126-17
Typhlocyba modesta | RRMFI2926-15 | Insecta | Hemiptera
SEQUEL | PBBCB001-17
Typhlocyba | RRMFI2845-15 | Insecta | Hemiptera
SEQUEL | PBBCB329-17
Typhlocyba pomaria | RRMFG2007-15 | Insecta | Hemiptera
SEQUEL | PBBCB639-17
Cicadellidae | RRMFI2741-15 | Insecta | Hemiptera
SEQUEL | PBBCB277-17
Typhlocybinae | RRMFG430-15 | Insecta | Hemiptera
SEQUEL | PBBCB553-17
Typhlocyba niobe | RRMFG419-15 | Insecta | Hemiptera
SEQUEL | PBBCB239-17
Paraphlepsius | RRMFI2736-15 | Insecta | Hemiptera
SEQUEL | PBBCB461-17
Graminella nigrifrons | RRMFI2837-15 | Insecta | Hemiptera
SEQUEL | PBBCB145-17
Planicephalus | RRMFI2767-15 | Insecta | Hemiptera
SEQUEL | PBBCB675-17
Eutettix variabilis | RRMFI4019-15 | Insecta | Hemiptera
SEQUEL | PBBCB855-17
Cicadellidae | RRMFI4037-15 | Insecta | Hemiptera
SEQUEL | PBBCB132-17
Colladonus clitellarius | RRMFI2933-15 | Insecta | Hemiptera
SEQUEL | PBBCB405-17
Latalus sayii | RRMFI2777-15 | Insecta | Hemiptera
SEQUEL | PBBCB570-17
Sorhoanus pascuellus | RRMFI2709-15 | Insecta | Hemiptera
SEQUEL | PBBCB035-17
Errastunus ocellaris | RRMFG2063-15 | Insecta | Hemiptera
SEQUEL | PBBCB187-17
Scaphoideus | RRMFC1362-15 | Insecta | Hemiptera
SEQUEL | PBBCB015-17
Scaphoideus major | RRMFD063-15 | Insecta | Hemiptera
SEQUEL | PBBCB931-17
Macrosteles quadrilineatus | RRMFE3202-15 | Insecta | Hemiptera
SEQUEL | PBBCB118-17
Macropsis fumipennis | RRMFI2750-15 | Insecta | Hemiptera
SEQUEL | PBBCB099-17
Macropsis basalis | RRMFG3167-15 | Insecta | Hemiptera
SEQUEL | PBBCB743-17
Oncopsis | RRMFG447-15 | Insecta | Hemiptera
SEQUEL | PBBCB801-17
Ceratagallia | RRMPB117-15 | Insecta | Hemiptera
SEQUEL | PBBCB581-17
Agallia quadripunctata | RRMPD001-15 | Insecta | Hemiptera
SEQUEL | PBBCB626-17
Limotettix ferganensis | RRMFI2734-15 | Insecta | Hemiptera
SEQUEL | PBBCB465-17
Limotettix | RRMFI2710-15 | Insecta | Hemiptera
SEQUEL | PBBCB665-17
Balclutha | RRMFG404-15 | Insecta | Hemiptera
SEQUEL | PBBCB432-17
Entylia carinata | RRMFD001-15 | Insecta | Hemiptera
SEQUEL | PBBCB364-17
Cyrtolobus vau | RRMFE012-15 | Insecta | Hemiptera
SEQUEL | PBBCB836-17
Atymna helena | RRMFE005-15 | Insecta | Hemiptera

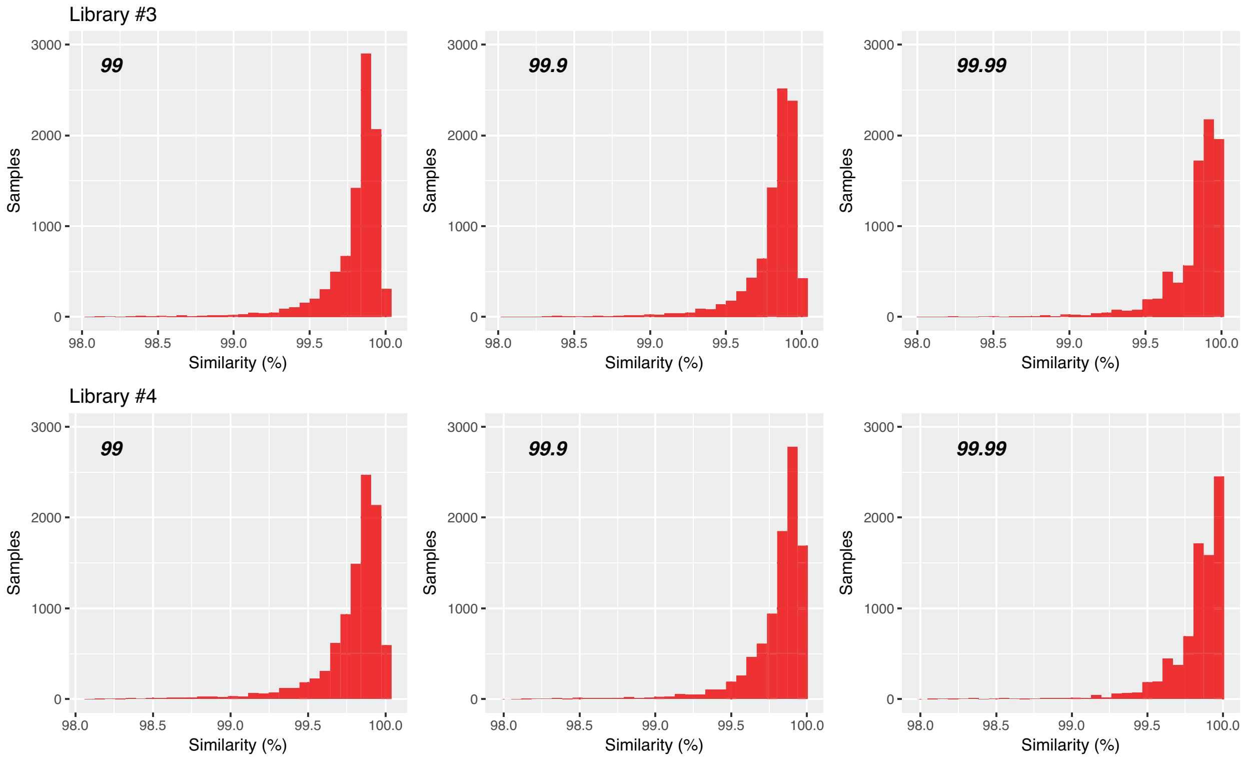

Figure S5. Distribution of similarity values between Sanger and SMRT sequences for the 9120 specimens in Library #3 and the 9830 specimens in Library #4 for all three SMRT partitions.

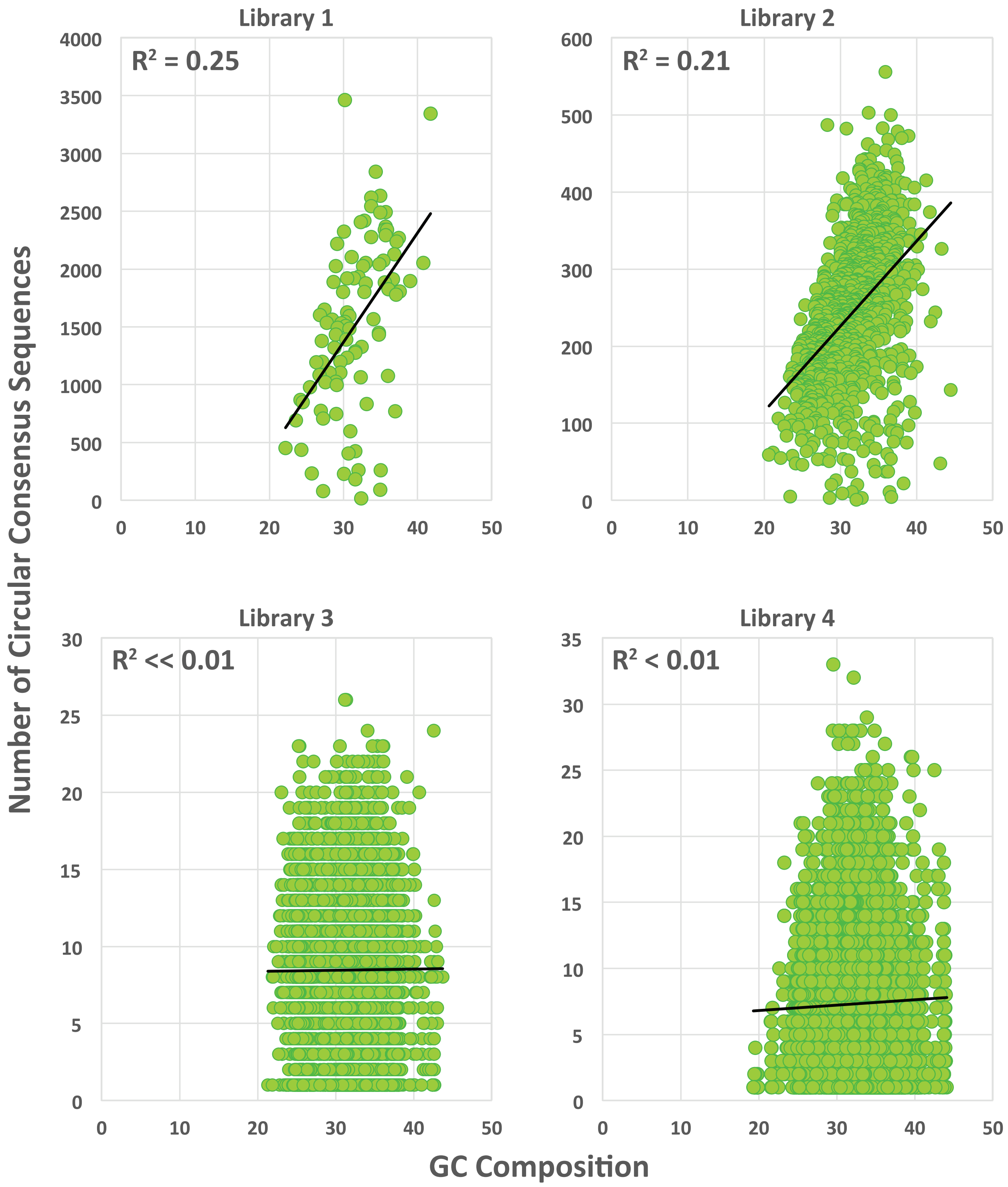

Figure S6. Relationship between the number of circular consensus sequences for a COI amplicon and its GC content. The 95 and 950 amplicon datasets (Libraries #1/2) were generated via the PCR1 protocol while the 9120 and 9830 amplicon datasets (Libraries #3/4) were generated via the PCR2 protocol.

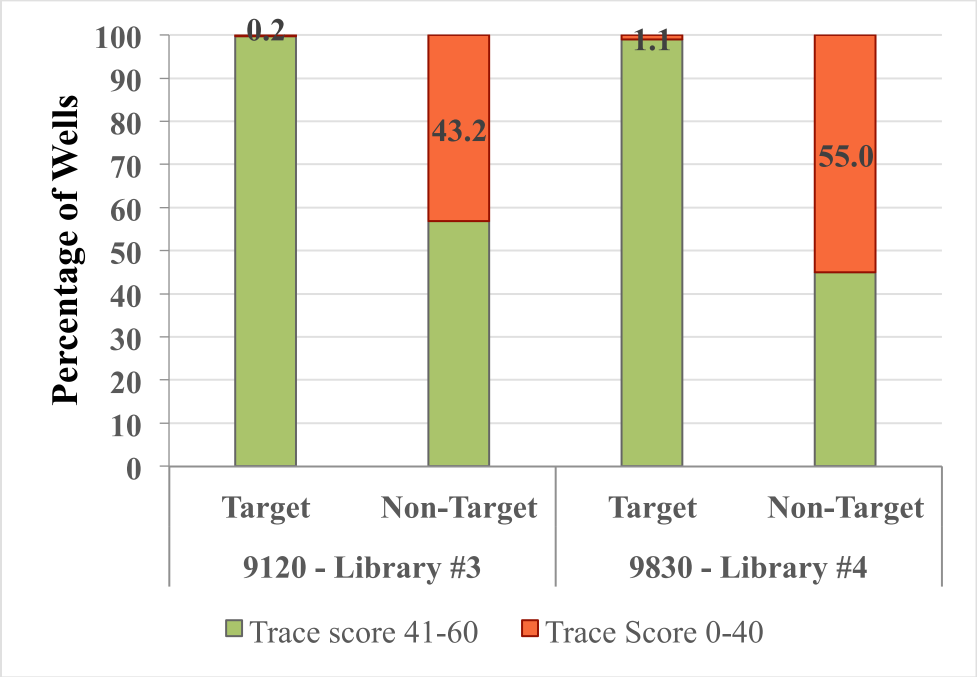

Figure S7. Incidence of low quality Sanger reads in cases of target and non-target wells for Libraries #3/4.
